# Potentiation of curing by a broad-host-range self-transmissible vector for displacing resistance plasmids to tackle AMR

**DOI:** 10.1371/journal.pone.0225202

**Published:** 2020-01-15

**Authors:** Alessandro Lazdins, Anand Prakash Maurya, Claire E. Miller, Muhammad Kamruzzaman, Shuting Liu, Elton R. Stephens, Georgina S. Lloyd, Mona Haratianfar, Melissa Chamberlain, Anthony S. Haines, Jan-Ulrich Kreft, Mark. A. Webber, Jonathan Iredell, Christopher M. Thomas

**Affiliations:** 1 Institute of Microbiology & Infection and School of Biosciences, University of Birmingham, Edgbaston, Birmingham, England, United Kingdom; 2 Institute of Microbiology and Infection, College of Medical and Dental Sciences, University of Birmingham, Edgbaston, Birmingham, England, United Kingdom; 3 University of Sydney, Centre for Infectious Disease & Microbiology, Westmead Institute of Medical Research, Westmead, New South Wales, Australia; University of Graz, AUSTRIA

## Abstract

Plasmids are potent vehicles for spread of antibiotic resistance genes in bacterial populations and often persist in the absence of selection due to efficient maintenance mechanisms. We previously constructed non-conjugative high copy number plasmid vectors that efficiently displace stable plasmids from enteric bacteria in a laboratory context by blocking their replication and neutralising their addiction systems. Here we assess a low copy number broad-host-range self-transmissible IncP-1 plasmid as a vector for such curing cassettes to displace IncF and IncK plasmids. The wild type plasmid carrying the curing cassette displaces target plasmids poorly but derivatives with deletions near the IncP-1 replication origin that elevate copy number about two-fold are efficient. Verification of this in mini IncP-1 plasmids showed that elevated copy number was not sufficient and that the *parB* gene, *korB*, that is central to its partitioning and gene control system, also needs to be included. The resulting vector can displace target plasmids from a laboratory population without selection and demonstrated activity in a mouse model although spread is less efficient and requires additional selection pressure.

## Introduction

**Fig 1:**
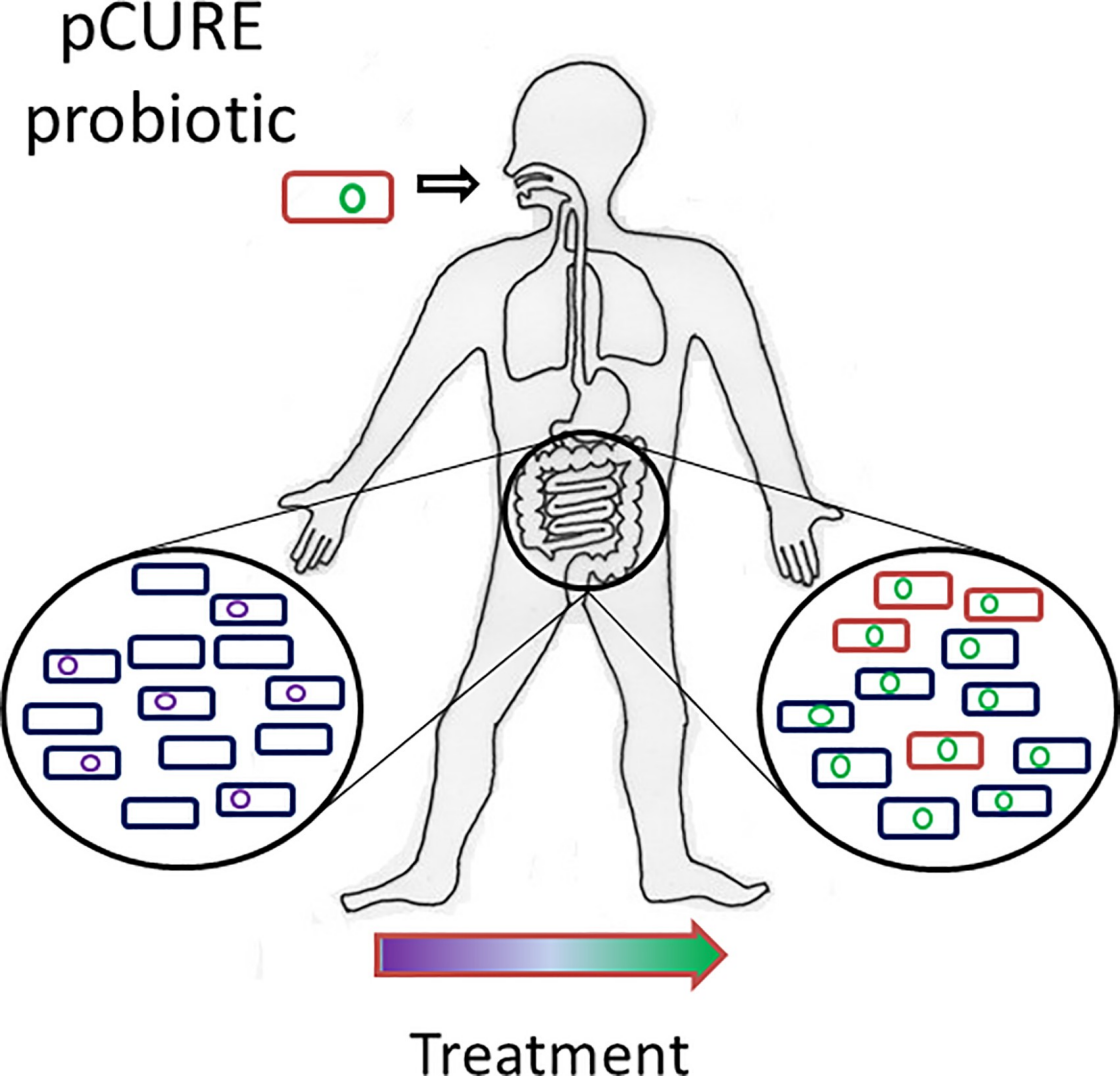
Anticipated exploitation of pCURE as a probiotic treatment for at-risk individuals. Before treatment, plasmids carrying antibiotic resistance genes are shown as purple circles. After treatment, target plasmids are replaced by pCURE (green circle) which could be engineered to later “self-destruct”. Note that not all gut bacteria carry resistance plasmids but pCURE can enter all Enterobacteriaceae as well as other bacteria. Reduced resistance levels in the gut decrease the chance of treatment failure when infections elsewhere in the body (eg lungs or urinary tract) arise from gut bacteria.

Antibiotic resistance in bacteria is an increasingly urgent problem that is one of the key global challenges to public health [1]. The rise of resistance is due to the selective pressure from widespread use of antimicrobial agents, combined with the genetic plasticity of bacteria, allowing resistance mechanisms to evolve and spread rapidly between bacteria. Once such resistance mechanisms exist, it is very difficult to get rid of them. Plasmids, which are relatively small, independently-replicating DNA elements, are a key part of the genetic arsenal of bacteria. Many plasmids can transfer between bacteria via conjugation and help bacteria to acquire advantageous genes from other bacteria [2]. In clinical contexts plasmids have often accumulated resistance determinants to all the antimicrobial agents that their hosts have been exposed to. One of the biggest clinical challenges is caused by Gram-negative bacteria resistant to carbapenems and third generation cephalosporin antibiotics due to plasmid-encoded enzymes. A possible way to reverse this situation is to displace the resistance plasmids themselves from reservoirs of resistance such as the gut [3] (Fig 1). Selectively removing plasmids, and the resistance genes they carry, from an in-situ population should avoid causing disruption to the commensal microbial community since this could have harmful consequences; for example, antibiotics eradicating normal gut microbiota can allow *Clostridium difficile* to overgrow with deadly effect in vulnerable patients [4].

Displacement of plasmids from bacteria, known as curing, commonly involves stresses such as increased growth temperature or limiting thymine [5]. Such treatments are not feasible in human or animal hosts, but specific interference with plasmid maintenance functions may be practicable. For stable inheritance, plasmids have evolved a variety of mechanisms: controlled replication, active partitioning, multimer resolution and “addiction” or post-segregational killing (PSK) systems [6]. PSK systems rely on expression from the plasmid of both an unstable antitoxin or regulatory RNA and a stable toxin or metastable RNA encoding delayed toxin expression: the toxin becomes active in the cell after plasmid loss [7].

When closely related plasmids are introduced into the same cell, they segregate into separate lineages as the cells divide, a phenomenon known as incompatibility [8]. This has been frequently used as a way to displace plasmids from bacteria. We previously used this approach, creating conjugative ‘curing plasmids’ by deleting the PSK toxin gene and adding a different selectable marker [9] thus creating an unstable plasmid that was incompatible with its target resistance plasmid. The introduced resistance marker allowed spread into the target population to be selected, causing displacement of the target plasmid. Once the target plasmid had disappeared, selection was removed allowing the curing plasmid to also disappear (due to its lack of functional addiction system) so that the host microbiome could recover. Unfortunately, because incompatibility between competing replicons is generally symmetrical, this approach depends on selection—which may affect bystander bacteria.

**Fig 2:**
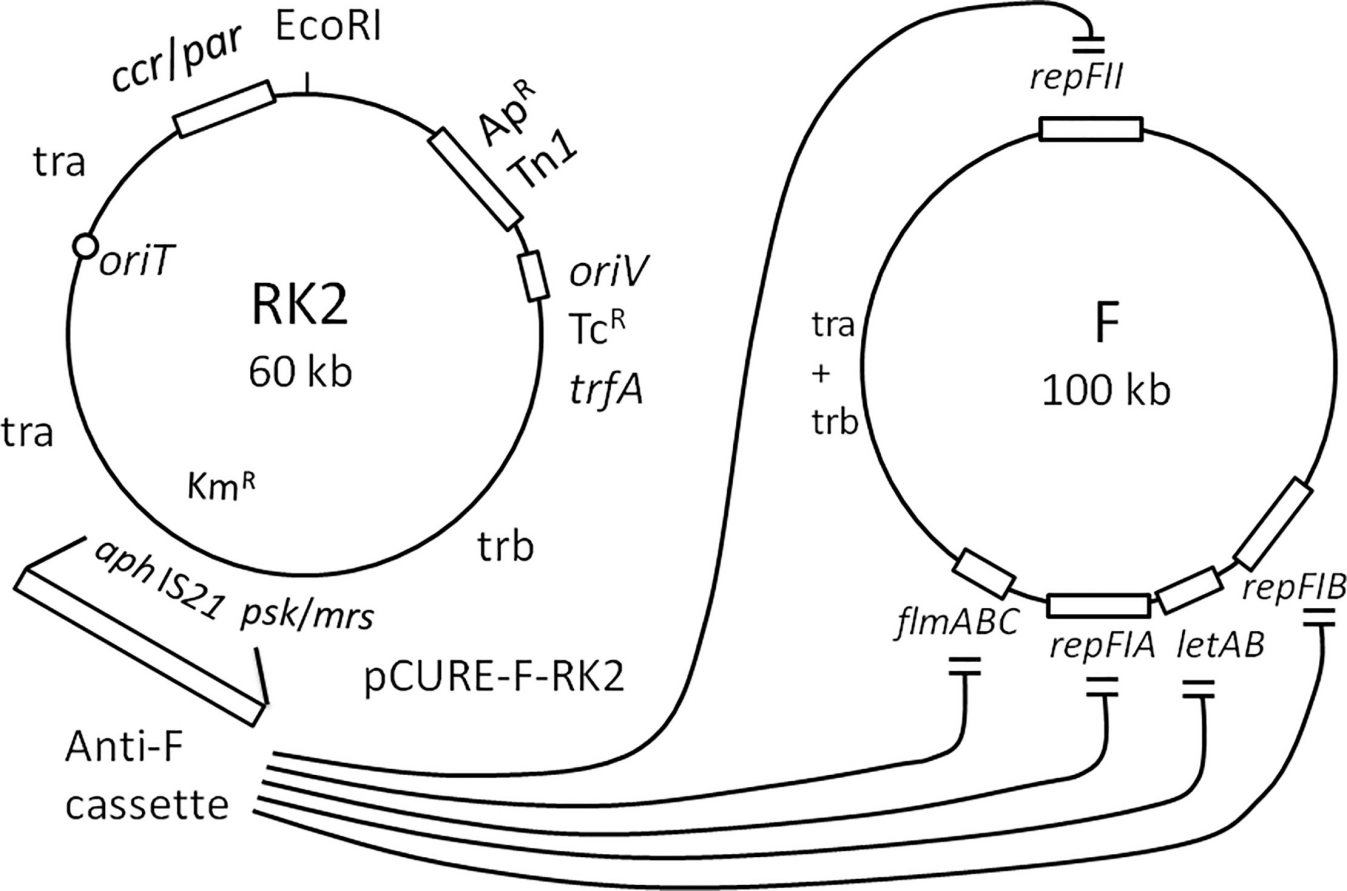
Map of RK2 showing region replaced by the anti-F cassette and showing the targets of the anti-F functions. The other functions marked are: *oriV*, the vegetative replication origin; *oriT*, the transfer origin; *tra* and *trb* regions encoding proteins for DNA processing and mating bridge formation during transfer; *trfA*, encoding the replication protein that activates *oriV*; ccr/par, the central control region that regulates transcription of RK2 backbone genes and also encodes active partitioning functions; psk/mrs, encoding addiction and multimer resolution functions. Mobile elements Tn*1* and IS*21* are associated with ampicillin and kanamycin resistance genes. Recombineering was used to replace *aphA* and IS*21* in RK2 with the anti-F cassette that blocks the repFIA, repFIB and repFII replicons and neutralises the effect of the *flmABC* and *letAB* addiction loci [12].

The work described here therefore uses a related, unidirectional approach where a reproductively unrelated vector displaces the target plasmid because it carries genetic regions which block all replication and addiction systems the target plasmid encodes. This is a complex task when target plasmids carry multiple replicons and PSK systems, as in the very common F-like plasmids of Enterobacteriaceae (Fig 2) [10,11, 12]. Efficient displacement of the F-like plasmids pO157 [13,14], p1658/97 [15] and pKDSC50 [16], as well as F′prolac [17] was previously achieved using a high-copy number vector compatible with the target plasmid [12]. Into this vector we inserted segments of the three F family replicons, FIA and FIB (encoding multiple binding sites or iterons that bind Rep protein and may “handcuff” the target plasmid) and FII (encoding the transcriptional repressor CopB and the anti-sense RNA CopA that blocks *repA* translation) to shut off F-like plasmid replication. We also added either the antidote or the repressor from relevant addiction systems to prevent them blocking growth and division of bacteria that lose the target plasmid. Together these anti-replication and anti-addiction segments form the “Anti-F cassette” [12]. This approach has since been extended to other plasmid groups, including IncI [18] and IncK (this work).

Our previous work used a vector that is mobilisable but not self-transmissible [12]. To displace resistance plasmids from the microbiota of complex environments such as a human or animal gut, a broad-host-range conjugative plasmid which can spread between many genera of bacteria may provide a solution. We therefore chose the well-studied IncP-1 Birmingham plasmid RK2 (essentially identical to RP1 and RP4) [19] as our starting point (Fig 2). IncP-1 plasmids, originally identified as responsible for the spread and maintenance of carbenicillin resistance in skin and gut bacteria [20,21], can transfer to both Gram negative and positive bacteria (even though they cannot replicate in Gram positives) [22]. Moreover, the copy number of RK2 has been determined as 3–7 per chromosome, which is higher than many large conjugative plasmids that would be prime targets for displacement, so cloned segments could be active in blocking their replication. Implementing this approach revealed that RK2 carrying the anti-F cassette does not displace target IncF or IncK plasmids efficiently but that removal of a previously uncharacterised control element from the replication origin can increase copy number and allows very efficient curing. Dissection of the curing plasmid also shows that the RK2 global regulator KorB that binds multiple places on RK2 is needed for efficient curing. The curing plasmids can spread and cure on solid medium without antibiotic selection but selection is still needed to achieve target plasmid loss from a mouse gut. The results will help define a strategy for building a successful conjugative pCURE plasmid.

## Results

### The anti-F cassette in RK2 displaces F plasmids inefficiently

**Fig 3:**
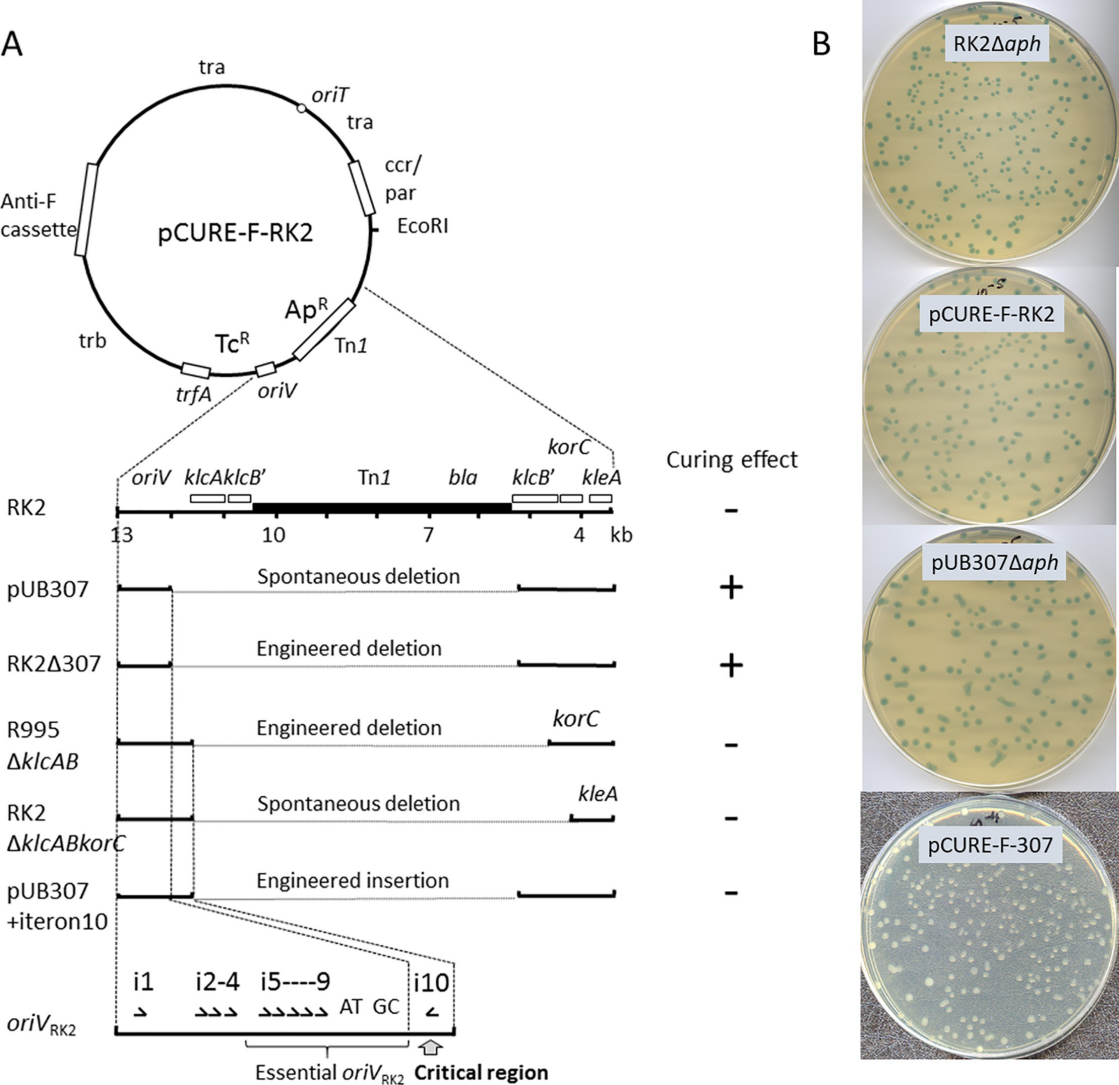
**Conjugative IncP-1 derivatives and their effectiveness as vehicles for plasmid curing (panel A).** The anti-F cassette inserted into RK2 caused limited displacement, as indicated by the blue/white X-gal+IPTG phenotype (compare RK2Δ*aph* and pCURE-F-RK2 in panel B), whereas when inserted into pUB307 (23), it causes very efficient F’ plasmid loss and no trace of blue colour (compare pUB307Δaph with pCURE-F-307 in panel B). Introducing the pUB307 deletion (bases 5464..5467 to 12045..12047) into RK2 potentiated curing while the deletions Δ*klcAB* and Δ*klcABC* (bases 4340–11669) did not. Reintroducing the region with i10 (bases 11749 to 12048) into pUB307 destroyed the potentiation, showing that this region includes the critical sequence.

The previously constructed anti-IncF cassette from pCURE2, designed to displace IncF plasmids, including loci that inhibit replication (repFIA, *incC*; repFIB; repFIC, *copAB*; repFIC/repFIIA, *copAB*) and that neutralise addiction systems (*flmB*, *sok*; *letA*, *ccdA*; *pemI*, *srnC*, *sok*) [12], was inserted into RK2 by recombination to replace the *aph* (Km^R^) gene (Fig 2) as described in Methods, creating pCURE-F-RK2 (Fig 3; nomenclature indicates first that it carries a curing cassette, second the plasmid group it targets, and third the plasmid it is based on). This plasmid, along with RK2 as a control, was tested for the efficiency with which it can displace F’prolac from *E*. *coli* JM109. This was detected by both loss of ability to grow on M9 medium without proline and by blue/white X-gal screening when JM109 carries a plasmid like pUC18, selectable with ampicillin, that complements the *lacZ* defect in the *lac* operon carried by the F’ so that the starting plasmid is phenotypically Lac^+^ (Fig 3 and Table 1).

**Table 1:** Curing by key conjugative anti-F and anti-K plasmids constructed in this study.

	Target plasmid and % cured ^a^			
Curing plasmid or control (ctl)	F’prolac initial^b^ colonies	F’prolac after^b^ re-culturing	pEK499 (IncF) initial colonies	R387 (IncK) initial colonies
**RK2 (vector ctl)**	**<1**	**<1**	**<1**	**<1**
**RK2Δ*aph* (vector ctl)**	**<1**	**<1**	**<1**	**<1**
**pCURE-F-RK2**	**<1**	**>85**	**<1**	**ND**^**c**^
**pUB307 (vector ctl)**	**<1**	**<1**	**<1**	**<1**
**RK2Δ307 (vector ctl)**	**<1**	**<1**	**ND**^**c**^	**ND**
**pCURE-F-307**	**>99**	**ND**^**c**^	**<1**	**ND**^**c**^
**pCURE-F-RK2Δ307**	**>99**	**ND**^**c**^	**ND**^**c**^	**ND**^**c**^
**pCURE-K-RK2**	**ND**^**c**^	**ND**^**c**^	**ND**^**c**^	**<1**
**pCURE-K-307**	**ND**^**c**^	**ND**^**c**^	**ND**^**c**^	**>99**
**pCURE-FEK499-307**	**>99**	**ND**^**c**^	**>99**	**ND**^**c**^

a. These tests were carried out a minimum of three times on separate occasions. Comparisons were done by replica plating 100 colonies. The phenotype was generally very clear: <1 means 0/100 colonies had lost the target plasmid; >99 means 100/100 had lost the target plasmid; >85 means we saw significant curing but there were always some colonies that retained the target plasmid and 85% cured was the lowest rate observed. Blue/white screening was also used and gave a clear cut difference between efficient and inefficient curing as shown in Fig 2.

b. Stage 1 involved screening transconjugant colonies from initial selection plates for complete loss of Pro^+^ phenotype. Stage 2 involved re-culturing from initial colonies into LB medium + tetracycline selective for RK2 or pCURE-F-RK2, growing O/N, plating on L-agar + tetracycline and then screening for retention of the Pro^+^ phenotype.

c. Not Done

With pCURE-F-RK2 none of the initial transconjugant colonies were completely free of F’prolac (Fig 3B) but after growing cultures from single colonies and a second round of screening, displacement of F’prolac increased to >85% while RK2 without the anti-IncF cassette as well as RK2Δ*aph* did not affect F’prolac stability at all (Fig 3 and Table 1). This indicates that the anti-IncF cassette, when inserted into the IncP-1 plasmid to give pCURE-F-RK2, is able to sustain the unidirectional displacement of F-like plasmids but works less efficiently than when carried by the high copy number (>40 copies per chromosome) pCURE2 plasmid [12] which is based on the pMB1 replicon.

### Removal of *oriV* segment with Rep binding site i10 potentiates curing

To investigate curing of resistance plasmids carrying β-lactamases we inserted the anti-F cassette into a spontaneous deletion derivative of IncP-1 plasmid RP1 (indistinguishable from RK2), pUB307 [23], that has lost the transposon Tn*1* that includes the *bla* gene conferring Ap^R^. We had previously determined the ends of this deletion as running from position 5464..5466 to 12045..12047 (there are three bases at the junction that could come from either side of the deletion) in the IncP-1 genome sequence (accession BN000925.1) [19, 24], removing the transposon as well as flanking backbone sequences (Fig 3) [24]. Unexpectedly, this new plasmid (pCURE-F-307) was very effective in causing displacement of F’prolac from JM109 (Fig 3 and Table 1). To check whether the potentiation of curing activity is due to the known deletion rather than to changes elsewhere in the plasmid, we recreated the pUB307 deletion starting from RK2, by recombineering as described in Methods. The same potentiation of curing was observed (Table 1; pCURE-F-RK2Δ307), confirming that the deletion in pUB307 is indeed responsible for this effect.

To explore the genetic basis for the potentiation we used plasmids with deletions that remove sub-segments of the region deleted in pUB307. When the anti-F cassette was inserted into plasmid pR9242 (a deletion derivative of IncP-1 plasmid R995, very similar to RK2), that has an in-frame fusion of the first six codons of *klcA* to the stop codon of *klcB* [25], no potentiation was seen (Fig 3). We also obtained a spontaneous deletion removing *klcA*, *klcB* and *korC* (RK2Δ4340–11669) leaving the *kleA* operon under control of the *klcAp* [25,26] and this gave curing efficiency similar to that of pCURE-F-RK2 (Fig 3). Thus, removal of all or part of the *klcA*, *klcB*, *korC* operon is not the reason for the potentiation of curing observed in pUB307.

The other segment deleted in pUB307 is adjacent to *oriV* and contains a single repeated sequence motif called an iteron (iteron 10, i10, Fig 3) that should bind the RK2 Rep protein, TrfA [27]. The role of i10 has not been determined but deletion of the single iteron (i1) on the opposite side of *oriV* increased copy number [28] so deletion of i10 may do so too, as predicted by Larsen & Figurski [29] and this may explain the potentiation of curing ability. When we reinserted the short region that contains i10, giving pUB307::i10, and then inserted the anti-F cassette (giving pCURE-F-307::i10) potentiation was lost (Fig 3). copy numberDetermination of kanamycin resistance conferred by the constitutively expressed *aph* gene in RK2 and pUB307 showed that removal of the region with i10 increased resistance about 2-fold (S1 Fig) consistent with increased copy number.

To determine whether the increased ability to cure is specific for F-like plasmids we inserted the IncK replication control region of archetypal plasmid R387 [30,31] as an anti-K cassette into both RK2 and pUB307 at the same location and tested for curing of R387. Once again, low efficiency curing was observed with pCURE-K-RK2 and high efficiency with pCURE-K-307 (Table 1). To check whether the potentiation is specific to delivery by conjugative transfer (which involves a single-stranded DNA intermediate) we introduced the plasmids by transformation and observed the same potentiation.

### IncP-1 global regulator KorB is also needed for efficient curing

**Fig 4:**
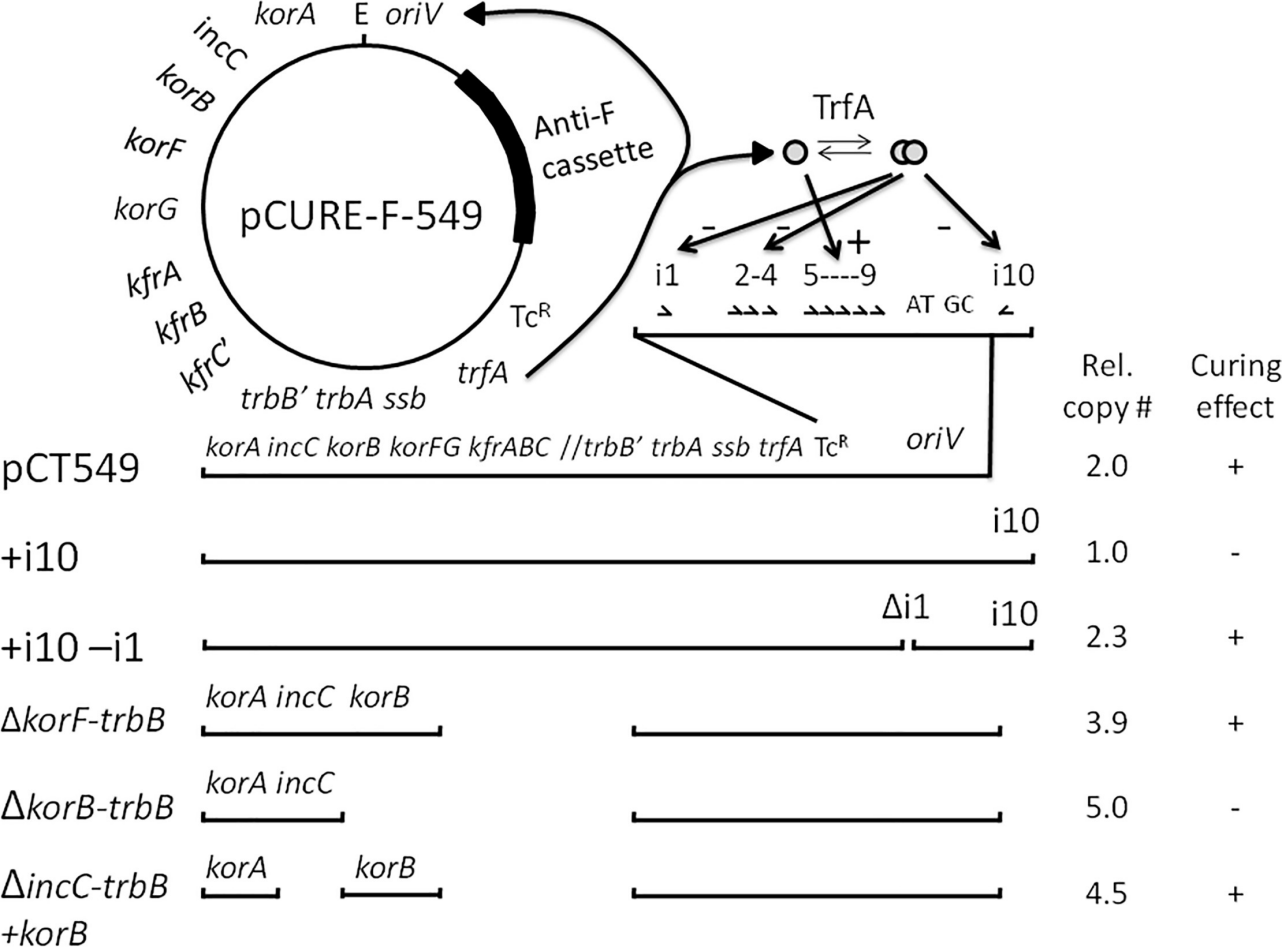
Mini-RK2 plasmids with the anti-F cassette and ability to displace F’prolac. Plasmid pCT549 consists of two segments from RK2: *oriV* to *trbB*’ and *korA* to *kfrC*’ (apostrophe indicates a truncated gene). TrfA acts at *oriV*: monomers act positively through iterons 5–9 and dimers act negatively through all the iterons. Due to its construction *oriV* of pCT549 excluded iteron 10 (i10) and with addition of the anti-F cassette efficient curing was seen. A derivative with iterons 1 to 10 gave a lower relative copy number (data and statistics in S2 Fig, S1 Raw Images and S1 Table) and efficient curing was lost. Deletion of i1 restored efficient curing and relative copy number rose approximately 2-fold. Deleting up to *korB* had no effect but deleting past it destroyed curing. Reinserting *korB* restored efficient curing.

Direct confirmation of a copy number difference associated with the presence/absence of i10 was obtained with mini-IncP-1 plasmid pCT549 [32] which, as constructed originally, does not have i10 (Fig 4). We amplified the *oriV* region from RK2 with i10 and substituted it into the pCT549 backbone. Comparison of plasmid DNA yield, with and without i10, using a compatible plasmid as internal control and nine samples per plasmid indicated that the copy number difference (statistically significant at <0.01 level in both an ordinary one tailed Student’s t-test for small samples and a one-tailed Welch’s t-test) was approximately 2-fold (Fig 4, S2 Fig, S1 Raw Images and S1 Table). This is consistent with the change in MIC for kanamycin between RK2 and pUB307. The same change in plasmid curing ability was observed in these mini-IncP-1 plasmids with anti-F cassette when the region with i10 was absent (Fig 4). We also considered the possibility that the change in potency could be due to some other property encoded in the region that contains i10 so with this region intact we deleted the region with i1 (bases 12928 to 12990 according to the complete IncP-1 backbone). This resulted in a similar rise in copy number and potentiation of curing, suggesting that removing either single iteron is sufficient for the change in curing activity (Fig 4).

As part of this analysis we also used as vector an even smaller derivative of RK2, pRK2501 [33] that does not include *korB* from the central control region and so is partially de-repressed for expression of *trfA* (the *rep* gene) and has a higher copy number (about 2.5 compared to pCT549 without i10)[32]. This did not support curing of the F’prolac from JM109 despite its elevated copy number, suggesting that at least one additional factor in the RK2 backbone may be necessary for the curing activity by the anti-F cassette in this context. We therefore deleted the major block of backbone genes in pCT549 that are not essential for replication and regulation–from the *trbB* promoter to the start of the *korF* gene (removing *kfrA*, *kfrB* and the remaining part of *kfrC* as well as *korF* and *korG*). This leaves just *oriV*, the *trfA* region (encoding the Rep protein TrfA) plus the central control/active partitioning region [34](encoding repressor KorA, partitioning ATPase IncC [a ParA homologue, the movement-generating component of the mitotic-like apparatus] and centromere-binding protein and global repressor KorB) and observed that this smaller plasmid still supports efficient curing.

Since the only major difference between this pCT549 derivative lacking the *korF* to *trbB* region and pRK2501 is the absence of a functional *korB* in pRK2501, it appeared that *korB* must be necessary for potentiation (Fig 4). We therefore made further deletions, up to the end of *incC* and up to the end of *korA* creating a deletion of all of *korB* and all of *incC2* (there are two translational starts in *incC*, the second of which overlaps the stop codon of *korA* and defines a shorter polypeptide product called IncC2) and these, as expected, give no curing. When we reinserted *korB* into the deletion derivative that had lost most of *incC* we found that this derivative had regained the ability to displace the target F plasmid efficiently (Fig 4). This indicates that the potent curing ability of an RK2–derived plasmid is not only dependent on manipulation of the *oriV* region but also requires an intact *korB* gene although a complete set of *par* functions is not essential (Fig 4).

### pCURE-307 spreads and displaces target plasmids without selection

**Fig 5:**
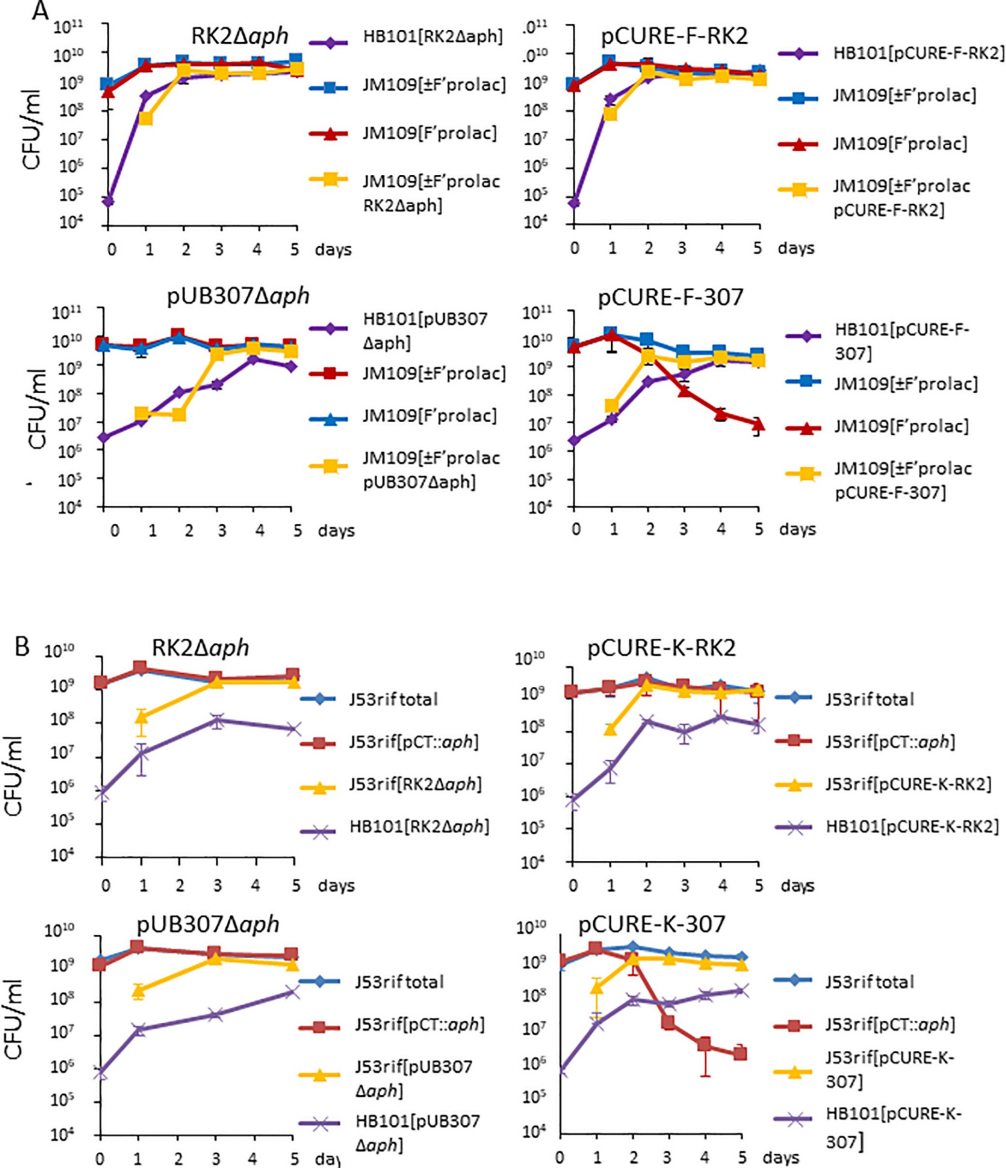
Unselected invasion assay to monitor displacement of target plasmids by pCURE plasmids or negative controls in bacteria introduced at a donor:recipient ratio of approximately 1:1000. A. Donor bacteria (HB101 with pCURE-F plasmids or negative controls) were monitored by streptomycin resistance, while target bacteria (JM109 initially with F’prolac) were monitored by nalidixic acid resistance. Presence of F’prolac was monitored on M9 Minimal medium without proline. The data is shown in the Tables in S1 Text. B. Donor bacteria (HB101 with pCURE-K plasmids or negative controls) were monitored by streptomycin resistance, while target bacteria (J53rif initially with pCT::*aph*) were monitored by rifampicin resistance. Presence of pCT::*aph* was monitored by kanamycin resistance. The data is shown in the Tables in S2 Text. Spread of pCT::*aph* into donor bacteria was detected by selection of kanamycin and streptomycin resistance as shown in the Figure in S3 Fig.

A critical test is whether pCURE can spread through a population and displace target plasmids without selection. Our initial test targeted the F’ plasmid in *E*. *coli* JM109 because it is easy to detect. Donor strains of *E*. *coli* (HB101 or MV10nal^R^ worked equally well) with control or test plasmids were mixed with *E*. *coli* JM109 at a ratio of 1 donor: 1000 recipients and 10^8^ bacteria of the mixture placed on a nylon filter on L-agar essentially as described by Fox et al. [35]. After overnight growth, bacteria on the filter were suspended in 2 ml saline and thoroughly mixed before spreading on a fresh nylon filter on fresh L-agar and serially diluting the bacterial suspension before spreading on selective and non-selective to determine plasmid carriage. This was repeated to give 5 cycles of growth and spread of pCURE. The results showed that pCURE plasmids spread rapidly and that pCURE-F-307 reduced the target F plasmid in a reciprocal way, with F’-positive bacteria falling to less than 0.1% of the target population (Fig 5A).

The F’ plasmid in JM109 is non-self-transmissible so we repeated this experiment with the self-transmissible IncK plasmid pCT::*aph* as the target in *E*. *coli* J53rif^R^ (Fig 5B). The results showed a similar spread of the pCURE plasmid and reduction of pCT::aph in J53rif^R^. In the RK2Δaph and pUB307Δaph controls pCT::*aph* also spread efficiently into the donor strain but the presence of both pCURE-K-RK2 and pCURE-K-307 prevented the formation of such transconjugant colonies, although there were some pinprick colonies where pCURE-K-307 was in the donor (S3 Fig). This was expected for pCURE-K-307 but was a surprise for pCURE-K-RK2, indicating that it is easier to stop a plasmid establishing itself than it is to get rid of it once established. Overall these results demonstrate that the elements for efficient curing are effective without selection.

### Extension of the anti-F cassette specificity

Since F-like plasmids are the commonest plasmid types encountered among multi-resistant Enterobacteriaceae and can also carry virulence functions [10,11], it is important that anti-F pCURE plasmids can be adapted to displace all possible targets. To test this we chose pEK499 [36] which we found was not displaced by the anti-F cassette in pCURE2 and pCURE-F-307 (Table 1). Bioinformatic analysis showed that the *copA* antisense RNA of pEK499 has 3 mismatches to the specificity loop in the repFII segment in the anti-F cassette which could be responsible for this. The *copAB* region of pEK499 was therefore amplified and incorporated into the anti-F cassette before it was inserted into pUB307 to give pCURE-FEK499-307. As predicted, the addition of this region allowed pEK499 displacement (Table 1). Thus the inability of pCURE-F-307 to displace pEK499 was due to the lack of activity against the FIC replicon and demonstrates the ease with which anti-F cassette specificity can be extended.

### Plasmid displacement in a mouse gut

**Fig 6:**
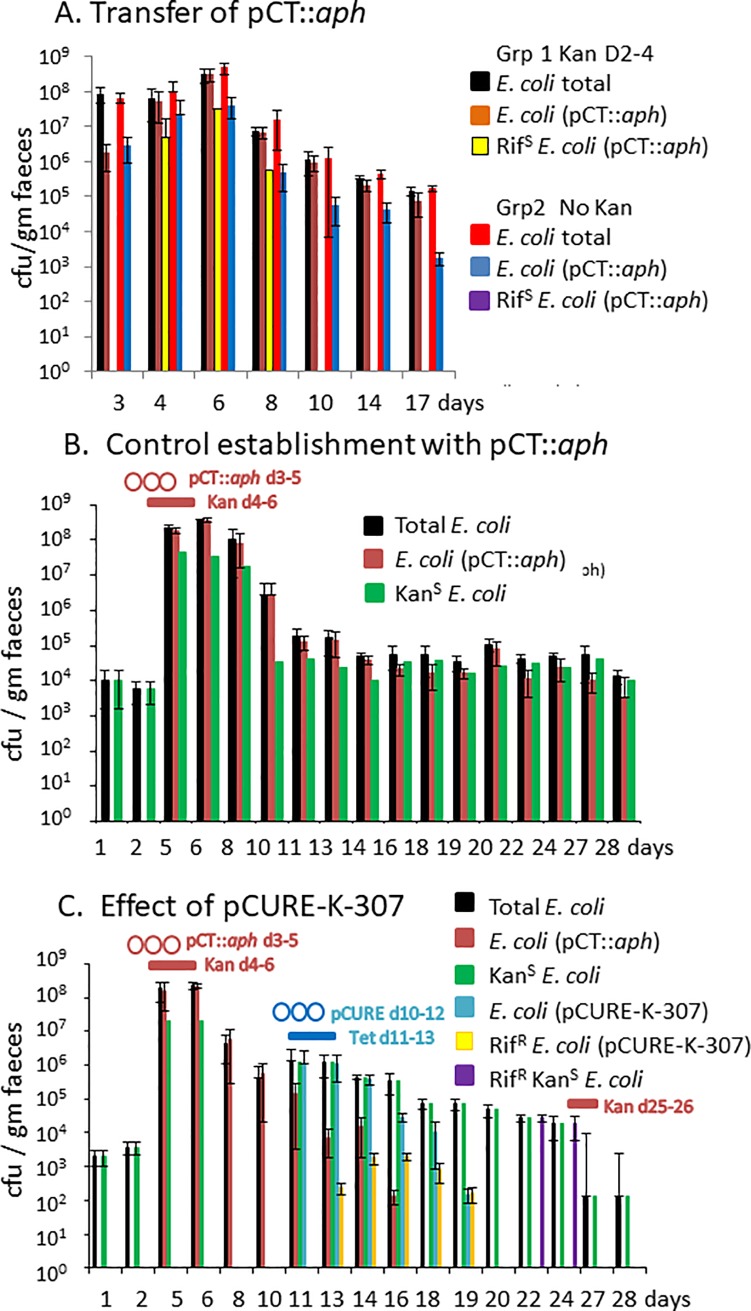
Testing ability of pCURE-F-307 to displace antibiotic resistance plasmids in the mouse gut. A. Rif^R^ mouse-derived *E*. *coli* AL1 carrying pCT::*aph* was fed to six mice days 1–3. Three mice (group 1) also received kanamycin days 2–4. Plasmid transfer to endogenous Rif^S^ *E*. *coli* was only observed in group 1. The data for this experiment is shown in the Tables in S3 Text. B. Baseline total endogenous Kan^S^Tet^S^ *E*. *coli* were detected before introduction of *E*. *coli* AL1 (pCT::*aph*) days 3–5 and kanamycin days 4–6 before monitoring plasmid carriage until day 28. Rif^S^ *E*. *coli* cfu were determined by subtracting Rif^R^ cfu from total *E*.*coli* cfu. The data for this experiment is shown in the Tables in S4 Text. C. Mice treated as in B received *E*. *coli* Nissle 1917 carrying Tet^R^ curing plasmid, pCURE-K-307 days 10–12 and tetracycline days 11–13 resulting in the appearance of Tet^R^ *E*. *coli* including Rif^R^ Tet^R^ indicating transfer to target bacteria. pCURE-K-307 had disappeared by day 20 but about 10% of the *E*. *coli* were Rif^R^ indicating displacement of pCT::*aph* rather than loss of the strain. Kanamycin was given days 25–26 but no Kan^R^ bacteria re-appeared and PCR screening of faeces samples at the end of the experiment proved negative. Carriage of pCT::aph was determined by Kan^R^ while carriage of pCURE-K-307 was determined by Tet^R^. The data for this experiment is shown in the Tables in S4 Text.

To determine whether our conjugative pCURE could spread without selection in an animal gut model we chose IncK plasmid pCT::*aph* as the target for displacement since it persists in diverse *E*. *coli* strains in different animals and humans [37]. It also possesses an active conjugative transfer system (in contrast to either the F’prolac or pEK499, neither of which are Tra^+^) thus representing a relevant *in vivo* challenge. The curing plasmid was pCURE-K-307 and the tests were performed in non-germ-free mice with normal gut microbiota to mimic a real-world situation but all mice were confirmed as being free of kanamycin and tetracycline resistant bacteria in their faeces before entering the experiment. After exploratory tests, we established bacteria carrying pCT::*aph* in the mouse by isolating an *E*. *coli* strain from the mice to be used. We selected a Rif^R^ mutant of this strain, and introduced pCT::*aph* into it (designated AL1) by conjugation and then fed AL1 (pCT::*aph*) to the mice as described in Materials and Methods. A preliminary experiment showed that the strain established itself very efficiently, reaching up to nearly 10^9^ Kan^R^ cfu g^-1^ faeces, constituting initially between 1 and 10% of the detectable *E*. *coli*. However, spread of pCT::*aph* to resident gut *E*. *coli* (which could be detected as Rif^S^) was not detected unless the mouse received kanamycin to select the plasmid starting the day after adding AL1(pCT::*aph*) (Fig 6A). That these strains were not simply mutants was confirmed by PCR with IncK-specific primers (see Methods). When feeding bacteria and antibiotic was stopped the number of *E*. *coli* decreased to about 10^6^ cfu g^-1^ faeces. In the main experiment (Fig 6B and 6C) in the control group of mice with no further treatment the level of Kan^R^ *E*. *coli* stabilised and decreased slowly for the rest of the experiment (Fig 6B), eventually becoming the minority of the *E*. *coli* detectable.

In the test group, once the level of pCT::*aph* was stable, we fed the mice with the *E*. *coli* Nissle1917 strain (which is accepted as a safe probiotic for human use; [38]) carrying pCURE-K-307. The temporary colonisation of the mouse gut by Nissle1917(pCURE-K-307) in the absence of selection was also good (we detected ~10^7^ Tet^R^ cfu g^-1^ faeces) although not as high as for mouse-derived strain AL1(pCT::*aph*). However, efficient transfer of pCURE-K-307 and displacement of pCT::*aph* was only detected in the group of mice that received Nissle1917(pCURE-K-307) with subsequent tetracycline for 3 days (Fig 6C). In the absence of any tetracycline selection the bacteria carrying pCURE-K-307 rose to about 10^6^ cfu g^-1^ faeces but transfer to the Rif^R^ *E*. *coli* already established was detected only transiently and at a low level (Panel A in S4 Fig). This was not increased by feeding *E*. *coli* Nissle1917(pCURE-K-307) for an extended period, although the Tet^R^ bacteria were shed for longer (Panel B in Figure in S4 Fig). In both cases it took about 6 days for bacteria with this plasmid to clear the mouse gut once the supply of bacteria to the mice was withdrawn.

Feeding *E*. *coli* Nissle1917(pCURE-K-307) followed by tetracycline resulted in a rapid decline and disappearance of Kan^R^ bacteria by day 18 followed by a decline in Tet^R^ bacteria (with pCURE-K-307). Superficially this was very encouraging but closer inspection indicates a complex situation. The possibility that tetracycline selection simply eliminated the bacteria lacking pCURE-F-307 was rejected by detection of Rif^R^Tet^R^ bacteria at day 13 and this number increased across days 14–16 before starting to fall again, suggesting that transfer was occurring in the absence of selection (Fig 6C). The proportion of the Tet^R^ bacteria that are Rif^R^ rises with time to nearly 100% (Fig 6C), suggesting that the donor strain disappears faster than the transconjugants, possibly because the donor is a human-adapted strain whereas the Rif^R^ bacteria are mouse-derived. This fits with disappearance of the Tet^R^ bacteria in S5 Fig, but persistence of the Kan^R^ bacteria even in the absence of selection (Fig 6A). However, the numbers of transconjugants where pCURE-K-307 has transferred into the Rif^R^ bacteria do not seem high enough to explain all of the decrease in pCT::*aph*. In the unselected curing experiments on agar (Fig 5A and 5B) the level of transconjugants with pCURE rose close to the original level of the target strain before curing was observed whereas in this experiment, *E*. *coli* (pCT::*aph*) is already dropping when still >10-fold higher than the Rif^R^ transconjugants with pCURE (Fig 6C). Although pCT::*aph* has also transferred to Rif^S^ *E*. *coli* and pCURE can also transfer to Rif^s^ *E*. *coli*, so some loss of pCT::*aph* may be from Rif^S^ *E*. *coli*, the numbers suggest that a major part of the drop in pCT::*aph* is likely due to the Tet treatment killing Kan^R^ Tet^S^ bacteria. Significantly though, 10 to 20% of bacteria isolated on days 22 and 24, when Kan^R^ and Tet^R^ bacteria had disappeared, were Rif^R^ indicating that an effective curing process has taken place, not just the elimination of the strain carrying pCT::*aph*.

That the target plasmid had disappeared completely was shown by a period of kanamycin selection on days 25–26 –this caused the *E*. *coli* counts to fall 100-fold but the remaining *E*. *coli* were still kanamycin sensitive. The data in Fig 6 is the average of three mice for each treatment so we also plotted the data for each individual mouse. Panels A, B and C in S5 Fig show the individual mouse data for the experiment in Fig 6C. This demonstrates that the average pattern observed is consistent with the observations at the individual mouse level and is remarkably reproducible. While the need for a period of antibiotic selection is not ideal, these results do demonstrate that curing plasmids are able to be delivered to the mouse gut and can transfer into target cells. The overall result is a gut that is free of the target bacteria and contains plasmid-free derivatives of the original plasmid-carrying bacteria. The use of a short selection with antibiotics allows endogenous microbiota to survive, leaving the potential to recolonise the gut.

## Discussion

This paper explores construction of an effective broad-host-range conjugative plasmid vector system to specifically displace target plasmids of different incompatibility groups, as an alternative strategy [39] to combat antimicrobial resistance. Although we previously explored such an approach by directly using incompatibility between two related plasmids [9], here we chose the well-studied IncP-1 plasmid RK2 as the basis for a more general vector to target plasmids compatible with RK2.

We first inserted our anti-F cassette that can target plasmids of the commonest group of conjugative plasmids in Enterobacteriaceae and found that wildtype RK2 is not a very effective vector for this displacement process. Although we are also trying to establish whether this is typical of other low copy number conjugative plasmids, we quickly discovered that a deletion-derivative removing a non-essential part of the extended replication origin region has a dramatically increased potency. This potentiation correlated with an approximately two-fold increase in copy number, resulting from loss of a region encoding a non-essential binding site (i10) for the replication protein TrfA. This iteron was only discovered when we compiled the whole IncP-1 plasmid sequence in the 1990s [19]: it was not included in the mini-derivatives constructed in the 1970s [40,28]. Larsen & Figurski [29] speculated that this iteron may work in copy number control alongside i1 (which we had shown to be an important control element; 28) since both have additional 6 bp repeats and are inverted relative to each other, suggesting loop formation that might impact negatively on *oriV* activity. The IncP-1 plasmid family is diverse, with at least six sub-families [41], but prompted by our findings, alignments of IncP-1 DNA sequences in Genbank show i10 is conserved across the IncP-1 sub-families (S6 Fig) implying its importance as part of the core system. Here we show that the region with i10 is part of the normal copy number control system with an effect equivalent to the region with i1 and that when this system is intact the plasmid is not a good vector for the curing cassettes, most likely because of its lower copy number. It also indicates that such copy number control should make this type of replicon highly evolvable by gain or loss of single iterons although clearly the configuration seen in RK2 has been conserved over considerable evolutionary time.

While setting vector copy number above a critical level may be important, we found that copy number alone is not sufficient for success. Finding that *korB*, but not an intact partitioning system, is also essential for potentiation of curing in the mini-RK2 plasmids, suggests that a critical factor may be the complexes that KorB protein makes with plasmid DNA and the effect they have on properties such as DNA topology. Unfortunately we cannot test whether this conclusion applies in the intact conjugative plasmid because *korB* is essential to regulate host-lethal functions on the plasmid [42] and thus cannot be deleted. KorB belongs to a broad family of DNA-binding proteins, many of which are involved in genome partitioning [43] and thus generically named ParB. Where structural studies have been carried out, it appears that ParB proteins bind, spread and condense DNA in a way that involves multiple cis- and trans- interactions [44,45]. In addition, it has recently been shown that ParB of *P*. *aeruginosa* binds to many places across its cognate genome [46], not just the well-recognised *parS* sites. ParB-family proteins may thus function more as Nucleoid-Associated Proteins (NAPs) than previously thought and modulate the topology of their cognate genome. Therefore, the binding sites for the replication protein TrfA and binding of KorB protein to RK2 DNA may need to be configured correctly to achieve a level of supercoiling sufficient to activate key loci of the anti-F and anti-K cassettes. Since the anti-K cassette is basically the antisense RNA regulatory elements of the IncK replicon from R387 [31], if there is just one part of the anti-F cassette that needs to be potentiated, it seems most likely to be the FII replicon [10,11], since this has the greatest similarity to the IncK replication control region. This in turn points to the possibility that the different components of a plasmid’s backbone maintenance functions may interact in a synergistic way to create the right genomic environment for optimal function of the plasmid’s regulatory circuits, a general point about plasmid organisation not previously recognised [6].

Much of the resistance gene load carried in otherwise healthy individuals is within the gut microbiota and plasmids carrying the resistance genes facilitate their dissemination throughout human communities and the global population as a whole [47]. Being able to displace plasmids from a target population was therefore a key goal of this work. Gratifyingly our pCURE based on pUB307 worked very efficiently on agar surfaces without selection so long as the bacterial community was remixed regularly in the way described by Fox et al [35]. The peristaltic action in the gut is effectively a remixing process which should aid transfer if effective mating-pairs between bacteria can occur. However, because it is well established that the thick, rigid pili encoded by IncP-1 plasmids are more suited to solid-surface mating [48] we anticipated a much greater challenge in an animal gut because this fluid or sludge-like environment is more complex and presents higher shear forces. This pessimistic view is in line with some published studies on the transfer of IncP-1 plasmids in the gut environment. For example, Licht et al (49) observed that although recipient and then donor strains could be established to high levels in the mouse gut while treating with sub-inhibitory concentrations of tetracycline, under the same regime, transfer did not occur at a detectable rate [49]. By contrast, comparison of loss rate of WT and a Tra^-^ mutant of IncP-1 plasmid pKJK5 indicate that its transfer system is important for retention in the rat gut, implying that significant transfer is occurring in the absence of selection [50]. This is consistent with our mouse experiments showing that once our donor strain was established by a period of selection, transconjugants could be easily detected and that their numbers rose at least ten-fold in the absence of selection while the donors disappear (Fig 6). The fact that our experimental regime did not first eliminate the natural microbiota by streptomycin treatment may play a role in the difference. This may also be encouraging for related use of IncP-1 plasmids such as the recent demonstration of RK2 as a vehicle for targeting plasmids with CRISPR-cas9 [51]. Indeed combining both approaches into a single plasmid might provide an even more powerful strategy for tackling antibiotic resistance in the future.

## Materials and methods

### Bacterial strains, plasmids and growth conditions

*Escherichia coli* strains used were DH5α [52], C600 [53], MV10Nal^R^ [54], JM109 [17], HB101 [55], Nissle1917 [56], AL1 (this study, Rif^R^ mutant of mouse *E*. *coli* strain isolated by Alessandro Lazdins in Sydney). Plasmids used are listed in the S2 Table. Bacteria were cultured aerobically, in either L-broth/L-agar [33], or M9 Minimal Medium [33] (supplemented with amino acids at 50 μg ml^-1^) at 37°C. Final antibiotic concentrations were: ampicillin (Ap or Amp), 100 μg ml^-1^; kanamycin (Km or Kan), 50 μg ml^-1^; chloramphenicol (Cm or Cam), 50 μg ml^-1^; nalidixic acid (Nal), 25 μg ml^-1^; rifampicin (Rif) 100 μg ml^-1^; and tetracycline (Tc or Tet), 25 μg ml^-1^. For the blue/white screening L-agar was supplemented with X-gal (20 μg ml^-1^) and IPTG (0.5mM).

### DNA analysis and manipulation

Restriction enzymes were purchased from New England Biolabs; T4 DNA ligase and Taq DNA polymerase were from Invitrogen; Velocity proof-reading DNA polymerase was from Bioline; Q5 high fidelity Taq polymerase was from NEB. PCR amplification of DNA was achieved using the primers (AltaBioscience, University of Birmingham, UK; or Sigma Aldrich) listed in S3 Table. Reactions were cycled in a SensoQuest Lab Cycler following standard procedures [57]. PCR products were purified using the Illustra GFX^TM^ PCR DNA and Gel Band Purification Kit (GE^TM^ Healthcare). Small-scale plasmid DNA preparations were performed using the AccuPrep Plasmid MiniPrep DNA Extraction Kit (Bioneer) adapted from the alkaline lysis method of Birnboim and Doly [58]. DNA sequencing reactions were prepared and run on an ABI 3730 DNA analyser (Functional Genomics Facility, University of Birmingham, U.K.) following the chain termination method [59].

### Comparison of plasmid copy number

At least triplicate selective overnight (16 h) cultures of *E*. *coli* carrying the query plasmids plus 2 kb pACYC194 derivative pDS3 as internal standard grown in LB with shaking at 200 rpm and 37°C were harvested and then plasmid DNA extracted as described above. Plasmid DNA was digested with an enzyme that would cut just once, to linearise the DNA and make ethidium bromide binding uniform. Band intensities were determined with QuantityOne software from Biorad and normalised against the pDS3 band. Colonies from serial dilution of the cultures were replica plated to determine % plasmid carriage.

### Conjugative transfer

Following overnight growth, 100 μl of donor was mixed with 1 ml of *E*. *coli* MV10 Nal^R^ recipient and filtered onto a 0.45 μm sterile Millipore filter. Filters were placed on L agar plates which were incubated at 37°C for 6 hours. Cells from the filter were resuspended in 1 ml of 0.85% (w/v) sterile saline solution each and serially diluted before spreading on selective agar and incubation at 37°C.

### Recombinational engineering (recombineering) of conjugative IncP-1 plasmid genomes

For insertions, deletions or replacements, primers (S3 Table) were used to PCR-amplify approximately 500 bp arms on either side of the point or region to be changed. The arms were joined by designing the internal primers to have complementarity, sometimes incorporating new restriction sites, to allow joining together by SOEing (Splicing by Overlap Extension) PCR [60]. Initial PCR products were purified, mixed and then extended for three cycles before adding the external primers for the remaining cycles. The product was routinely cloned between HindIII and SalI sites of a pACYC184 derivative pMEL1 that has the *sacB* gene (allowing counter-selection with sucrose) inserted between the XbaI and HindIII sites. To incorporate the antiF and antiK cassettes into a conjugative plasmid the homology arms were first inserted to give pMILL1 (S2 Table) and then the cassettes cloned between the arms (using restriction sites designed in the inner primers) to give pMILL2 and pSLK1 respectively (S2 Table).

The recombineering plasmid was introduced into *E*. *coli* C600 already carrying the target plasmid, selecting with Cam and an antibiotic appropriate for the target plasmid (routinely Tet). Conjugative transfer to MV10nal^R^ was then carried out selecting both markers and Nal–the pMEL1-derived plasmid should not transfer unless it has recombined with the conjugative plasmid. Individual colonies were purified by streaking to single colonies and these plates were used to inoculate liquid cultures which were grown overnight without Cam. Selection of resolution products was then achieved either by spreading on L-agar with sucrose (5% w/v) or by isolating plasmid DNA and cutting with XbaI that cuts in pMEL1 but not in the IncP-1 backbone to linearise the unwanted plasmids so that they will not transform bacteria.

### Testing curing efficiency

For transfer by conjugation, overnight liquid cultures of *E*. *coli* C600 carrying the pCURE plasmid to be tested, or an appropriate control, were washed to remove any selective antibiotics, then mixed 1:1 and 1:10 with a strain carrying the target plasmid and a standard filter mating carried out at 37°C for 1 h. Bacteria from the membrane were then resuspended in 1 ml sterile saline (0.85% w/v) and serially diluted before plating on selective agar to determine the total number of transconjugants and transconjugants still carrying the target plasmid. Displacement of F’*prolac* was determined in two ways: first, by spreading on M9 medium supplemented appropriately with and without proline to determine % loss of Pro^+^ phenotype; second, by growth on L-agar with IPTG and X-gal when the target strain additionally carried pUC18 (selected for with Amp) to give a Lac^+^ phenotype due to the presence of the lacZ–fragment encoded by pUC18, so that loss of F*’prolac* gave a Lac^-^ phenotype. When introducing the curing plasmid by transformation the target bacteria were made competent by standard CaCl_2_ treatment and transformation was done with purified pCURE DNA.

The unselected invasion assay was carried out by repeated mixing on nylon membranes as described by Fox et al., [35]. Donor bacteria were mixed with target strain to give approximately 10^6^ donors and 10^9^ recipient bacteria before spreading 100 μl on a nitrocellulose filter (25mm diameter, 0.45μm pore size, EMD-Millipore, Darmstad, Germany) on an L-agar plate. After 24 h incubation at 37°C the bacteria were resuspended in 1 ml saline, mixed thoroughly and serially diluted to determine both transfer and curing. The undiluted suspension (100 μl) was spread on a fresh nylon membrane on an L-agar plate to start the next mating period.

### Manipulation of mini-RK2 plasmids

Making pCT549 derivatives was awkward because insertion of EcoRI-BglII *oriV* fragments or BglII-PacI fragments into this plasmid proved difficult. To insert EcoRI-BglII *oriV* fragments without the antiF cassette the *oriV* segment was generated by PCR, joined to pGEM-T Easy and sequenced. The pGEMT-derivative and pCT549 were then cut with BglII and ligated before transformation into *E*. *coli* C2110, which is DNA polI deficient so does not allow replication of the pMB1 replicon in the pGEM-T, thus selecting cointegrants which also contain the IncP replicon that is PolI independent. Plasmid DNA from transformants was checked for the relative orientation of the joined segments and the one chosen that could be cut with EcoRI and recircularised by ligation to replace the old *oriV* with the new one.

To insert the antiF cassette beside *oriV*, primers were designed to put XbaI + EcoRI sites downstream of *oriV* and MfeI + PacI sites upstream, where BglII is normally. After cloning the MfeI-XbaI *oriV* fragment in pGEM-T Easy and checking the sequence it was ligated with the pACYC184 derivative pLAZ2 that had been cut with EcoRI and XbaI. In pLAZ2 EcoRI defines one end of the antiF cassette and the XbaI site is on the same side separated by one homology arm and the *sacB* gene. The other end of the antiF cassette in pLAZ2 is defined by a BglII site. The MfeI/EcoRI ends can join but do not regenerate either site so this construction generates a BglII-antiF-PacI-*oriV*-EcoRI-XbaI segment and this was inserted into pCT549 as described above involving BglII cutting, ligation and transformation into C2110. The antiF cassette was similarly inserted into mini-RK2 plasmid pRK2501 that already lacked *korB*.

To remove parts of the *korA*-*incC*-*korB*-*korF*-*korG*-*kfrABC* region and remnants of *trbB* near *trfA*, inverse PCR was carried out on pCT549+antiF cassette with primers incorporating an XbaI site since XbaI does not cut the RK2 backbone or the antiF cassette. Long range PCR was carried out with Q5 high fidelity Taq polymerase using their recommended primer design and conditions removing *korF*-*trbB*, *korB*-*trbB* and *incC*-*trbB*. The product was purified, cut with XbaI, recircularised and transformed into DH5α. To remove *incC* but not *korB* the *korB* orf was amplified and it plus the Δ*korF*-*trbB* plasmid were ligated after cutting with XbaI (cuts upstream of *trbB*p) and SphI which cuts in *incC*.

### Mouse experiments

All animal care procedures and experiments were approved by the Animal Ethics Committee of Western Sydney Local Health District (protocol 4276.08.17) in accordance with the ‘Australian Code of Practice for the Care and Use of Animals for Scientific Purposes’ and carried out essentially as described previously [9]. Five week old female BALB/c mice (Animal Resource Centre; Perth, WA, Australia) were housed in groups of three in open-lid M1 polypropylene cages (Able Scientific, Australia) on a 12 h light/dark cycle, with food and water available ad libitum (Biological Services Facility, Westmead Institute for Medical Research). Each different treatment involved groups of 3 mice. Mice were acclimatised (d-6 to d0) before experiments, followed by run-in (d1-d3) in the experimental room to establish the baseline. Mice were fasted for 6 h before being given access to sucrose water and then normal food was continuously available. Bacterial cultures carrying a plasmid were resuspended in sucrose water (8%, w/v) to an OD600 of ~0.4±0.05 and fed to mice on specified days and / or antibiotics (10–50 mg L^-1^) as appropriate. On the specified days each mouse was briefly transferred into a separate plastic box for weighing and to collect fresh faeces. Faeces (100 mg per mouse) were suspended in 1 ml phosphate buffered saline (PBS), dilutions plated on CHROMagar with appropriate antibiotics and colonies counted after incubation O/N at 37 ºC. Periodically 100 colonies were picked onto further plates to determine accurate proportions of resistance phenotypes. PCR to detect the IncK plasmid replicon in Rif^S^ *E*. *coli* was done using primers AL_IncK_F and AL_IncK_R (S3 Table). Mouse faeces solutions (100 mg in 1 ml saline) were diluted 1:100 times in water and 3 μl used as template in 50 μl reaction and *E*. *coli* carrying pCT::*aph* was used as positive control. At the end of the experiment PCR was carried out to determine whether any pCT::*aph* plasmid DNA could be detected if that was not evident by direct plating. Mice were euthanised by an overdose of CO_2_ immediately after completion of experiments.

The groups of mice were as follows. **Group 1** (control group): received normal food and drink plus sucrose water when other groups received it but without bacteria or antibiotics. **Group 2** (antibiotic control group): received normal food and drink plus sucrose water with antibiotics when groups 3 & 4 received antibiotics. **Group 3** (colonisation control group): received *E*. *coli* AL1 (pCT::*aph*) in sucrose water for days 3–5 plus Kanamycin for days 4–6, then normal food and water for the rest of the experiment, monitoring *E*. *coli* AL1(pCT::*aph*) in faeces until end of experiment. **Group 4** (curing experimental group A): received *E*. *coli* AL1(pCT::*aph*) in sucrose water for 3 days plus Kan (3 days) as group 3, then challenged with *E*. *coli* Nissle1917(pCURE-K-307) for days 10–12 and Tet for days 11–13 and monitored faeces for different sets of *E*. *coli* (endogenous coloniser, curing strains and challenger). **Group 5 (**curing experimental group B): received *E*. *coli* AL1 (pCT::*aph*) in sucrose water for 3 days plus Kan (3 days), then challenged with *E*. *coli* Nissle1917 (pCURE-K-307) for 3 days but no antibiotics and monitored faeces as above. **Group 6** (curing experimental group C): received *E*. *coli* AL1 (pCT::*aph*) in sucrose water for 3 days plus Kan (3 days), then challenged with *E*. *coli* Nissle1917 (pCURE-K-307) every day for 8 days (d10-17) and monitored faeces as above.

## Data and material availability

Data for all experiments can be obtained from the corresponding author. Curing plasmids can be purchased from Plasgene via the corresponding author on receipt of a signed Material Transfer Agreement for non-commercial use only. Other materials are available on request from the corresponding author.

## Supporting information

S1 Table Copy number comparison for plasmids shown in Fig 4 and S2 Fig. (DOCX)

S2 Table Plasmids used and constructed during this study. (DOCX)

S3 Table Primers designed and used during this study. (DOCX)

S1 Fig Growth of *E. coli* C600 with different plasmids at increasing concentrations of kanamycin. A) Comparison of kanamycin resistance of C600, C600[RK2] and C600[pUB307]. C600[RK2] has an MIC of 600μg ml^-1^, whereas C600[pUB307] is giving an MIC of 1000–1200μg ml^-1^. B) Comparison of kanamycin resistance of the same strains as tested in panel A but including additional plasmids. C600[pUB307] is still showing higher resistance than C600[RK2] while C600[pUB307::i10] gives resistance to levels of kanamycin (400–800μg ml^-1^) more similar to RK2 than to pUB307. C600[RK2𠎔4340–11669] also gives similar resistance to RK2.
(DOCX)

S2 Fig Comparison of plasmid copy number for key plasmids after curing with EcoRI. A. All plasmids compared in triplicate. B. Six isolates pCT549 and pCT549+i10 compared in pairs, overexposed to make the RK2-derived plasmid bands more visible. Band intensities were determined using Quantity One software after adjusting the exposure to ensure that the image was not saturated and then normalised by taking the ratio to the control pDS3 band–numerical data is shown in S1 Table. To give maximum confidence in the pCT549 v pCT549+i10 comparison these two plasmids were also compared alone with multiple samples paired to ensure identical conditions. S1 Table shows the signal for each band after subtracting background. Means and standard deviations were calculated and statistical analysis performed for the critical pCT549 v pCT549+i10 comparison. S1 Raw Images shows the uncropped images used in this Figure.
(DOCX)

S3 Fig Invasion of the donor strain in the unselected invasion assay with pCT::*aph* as the target plasmid. Both HB101 and MV10nal^R^ were successfully used as *E*. *coli* donor host strains. Transfer into the donor in the experiment with MV10nal^R^ as the host was detected by selecting resistance to nalidixic acid for the host and kanamycin for the plasmid. Mating mixtures were re-suspended in saline and after serial dilution 20 μl aliquots were spotted in a circle round the plate. The numbers are low so it was not easy to plot them on the same log scale as the data shown in Fig 5.
(DOCX)

S4 Fig Effect of pCURE-K-307on carriage of pCT::*aph* in the absence of selection with tetracycline for comparison with the data in Fig 6C and S5 Fig. The key points are that the frequency of Rif^R^ *E*. *coli* (pCURE-K-307) is very low and that pCT::*aph* persists throughout the experiment. The data is shown in the Tables in S4 text.
(DOCX)

S5 Fig Effect of pCURE-K-307 on presence of target plasmid pCT::*aph* in bacteria in the mouse gut when accompanied with a short period of tetracycline treatment after pCURE-K-307 was administered. In the mouse experiments three mice were used for each treatment and the mean at each time point plotted but the data in this Figure is for each individual mouse that was used to generate Fig 6C. The data is shown in the Tables in S4 text.
(DOCX)

S6 Fig Alignment of the *oriV* regions of plasmids representing IncP-1 subgroups α (RK2, BN000925.1), β (pRWC72a, JX486125.1; R751, NC_001735.4), γ (pQKH54, AM157767.1), δ (pEST4011, NC_005793.2) and ε (pKJK5, AM261282.1) to show the conservation of Iteron 10 in all five groups. The initial alignment was constructed in Clustal Omega but then optimised manually since the sequence divergence, particularly for pQKH54, makes some of the initial alignments unreliable. The Iteron consensus for pQKH54 is also slightly different from that of the other plasmids. Plasmid pRWC72a was chosen as the IncP-1β plasmid because it is like the other plasmids chosen in not having addition system genes between the *klcA* promoter and Iteron 10. The numbers at the right hand end of the lines refer to the coordinates in the Genbank files for these plasmids. The sequence is not extended as far as Iteron 1 because the size of the Figure then becomes too cumbersome.
(DOCX)

S1 Text Data for displacement of F’*prolac* shown in Fig 4A. (DOCX)

S2 Text Data for displacement of pCT::*aph* shown in Fig 4B. (DOCX)

S3 Text Data for the mouse experiment shown in Fig 6A. (DOCX)

S4 Text Data for the mouse experiments shown in Fig 6B and 6C as well as in S4 Fig and S5 Fig. (DOCX)

S1 Raw Images Raw (uncropped) images of the gels shown in S2 Fig. (PDF)
