# Supplementary material for: Potentiation of curing by a broad-host-range self-transmissible vector for displacing resistance plasmids to tackle AMR
Source: PLoS One. 2020 Jan 15;15(1):e0225202. doi: 10.1371/journal.pone.0225202 (PMC6961859; doi:10.1371/journal.pone.0225202)
Supplement: S1 Table — (DOCX) [file pone.0225202.s001.docx]

**S1 Table. Copy number comparison for plasmids shown in Figure 4 and Figure in S2_Fig.**

| Plasmid | Volume-background | Ratio | Mean±STD | Relative copy number |
| --- | --- | --- | --- | --- |
| pCT549 | 3135 | 0.14 |  |  |
| pDS3 | 22082 |  |  |  |
| pCT549 | 2962 | 0.12 | 0.137±0.039 | 2.07*** |
| pDS3 | 25691 |  | (variance = 0.0015) | (t test: t = 4.36 |
| pCT549 | 2906 | 0.13 |  | so p<0.01) |
| pDS3 | 22940 |  |  |  |
| pCT549 | 2205 | 0.10 |  |  |
| pDS3 | 21224 |  |  |  |
| pCT549 | 1543 | 0.095 |  |  |
| pDS3 | 16175 |  |  |  |
| pCT549 | 2345 | 0.098 |  |  |
| pDS3 | 22881 |  |  |  |
| pCT549 | 3619 | 0.18 |  |  |
| pDS3 | 20437 |  |  |  |
| pCT549 | 3207 | 0.17 |  |  |
| pDS3 | 18447 |  |  |  |
| pCT549 | 2476 | 0.20 |  |  |
| pDS3 | 12580 |  |  |  |
| pCT549+i10 | 716 | 0.045 | 0.066±0.031 | 1.00*** |
| pDS3 | 15817 |  | (variance = 0.00094) |  |
| pCT549+i10 | 1153 | 0.056 |  |  |
| pDS3 | 20454 |  |  |  |
| pCT549+i10 | 1274 | 0.066 |  |  |
| pDS3 | 19305 |  |  |  |
| pCT549+i10 | 1975 | 0.097 |  |  |
| pDS3 | 20345 |  |  |  |
| pCT549+i10 | 2211 | 0.12 |  |  |
| pDS3 | 18229 |  |  |  |
| pCT549+i10 | 1522 | 0.083 |  |  |
| pDS3 | 18377 |  |  |  |
| pCT549+i10 | 524 | 0.028 |  |  |
| pDS3 | 18760 |  |  |  |
| pCT549+i10 | 887 | 0.029 |  |  |
| pDS3 | 30191 |  |  |  |
| pCT549+i10 | 1776 | 0.066 |  |  |
| pDS3 | 26727 |  |  |  |
| pCT549+i10-i1 | 3103 | 0.16 | 0.15±0.01 | 2.27 |
| pDS3 | 19849 |  |  |  |
| pCT549+i10-i1 | 3284 | 0.14 |  |  |
| pDS3 | 22704 |  |  |  |
| pCT549+i10-i1 | 3357 | 0.15 |  |  |
| pDS3 | 22869 |  |  |  |
| pCT549ΔtrbA-korF | 2447 | 0.19 | 0.17±0.02 | 2.58(3.88) |
| pDS3 | 13101 |  |  | (size adjusted) |
| pCT549ΔtrbA-korF | 3298 | 0.15 |  |  |
| pDS3 | 21930 |  |  |  |
| pCT549ΔtrbA-korF | 2431 | 0.16 |  |  |
| pDS3 | 15507 |  |  |  |
| pCT549ΔtrbA-incC | 2158 | 0.18 | 0.19±0.006 | 2.87 (4.96) |
| pDS3 | 11876 |  |  | (size adjusted) |
| pCT549ΔtrbA-incC | 3442 | 0.19 |  |  |
| pDS3 | 18335 |  |  |  |
| pCT549ΔtrbA-incC | 3463 | 0.19 |  |  |
| pDS3 | 18490 |  |  |  |
| pCT549ΔtrbA-incC+korB | 3355 | 0.18 | 0.19±0.006 | 2.87(4.46) |
| pDS3 | 18328 |  |  | (size adjusted) |
| pCT549ΔtrbA-incC+korB | 3286 | 0.19 |  |  |
| pDS3 | 17276 |  |  |  |
| pCT549ΔtrbA-incC+korB | 3660 | 0.19 |  |  |
| pDS3 | 18786 |  |  |  |
| pRK2501 | 4403 | 0.40 | 0.27±0.11 | 4.09(5.76) |
| pDS3 | 10931 |  |  | (size adjusted) |
| pRK2501 | 3858 | 0.22 |  |  |
| pDS3 | 17224 |  |  |  |
| pRK2501 | 3678 | 0.19 |  |  |
| pDS3 | 19079 |  |  |  |
