# Supplementary material for: Potentiation of curing by a broad-host-range self-transmissible vector for displacing resistance plasmids to tackle AMR
Source: PLoS One. 2020 Jan 15;15(1):e0225202. doi: 10.1371/journal.pone.0225202 (PMC6961859; doi:10.1371/journal.pone.0225202)
Supplement: S2 Table — (DOCX) [file pone.0225202.s002.docx]

**S2 Table. Plasmids used and constructed during this study**

| **Plasmid** | **Properties** | **Reference** |
| --- | --- | --- |
| RK2 | IncP-1α; Amp^R^ Kan^R^ Tet^R^ | 21; Fig. 2, 3 |
| pUB307 | IncP-1α; Kan^R^ Tet^R^; spontaneous deletion that lost Tn1 | 23; Fig. 3 |
| pR9242 | IncP-1α (R995); Kan^R^ Tet^R^; site-directed deletion of KlcA Tn1 | 25 |
| F’*prolac* | RepFIA RepFIB RepFII Tra^-^ in JM109 | 17 |
| pCURE2 | pMB1 replicon, *oriT*RK2, *sacB*, anti-IncF; Amp^R^ Kan^R^ | 12 |
| pEK499 | IncF Tra^-^ Amp^R^ Sm^R^ Su^R^ Cam^R^ Tet^R^ Cp^R^ Tp^R^ | 36 |
| R387 | IncK Tra^+^ Cam^R^ Sm^R^ | 30 |
| pCT::*aph* | IncK Tra^+^ Kan^R^ | 37 |
| pDS3 | P15A replicon, Cam^R^ | Thomas (1981)^1^ |
| pMEL1 | From pACYC184: p15A replicon; Cam^R^, Tet^R^, *sacB* | This study |
| pMILL1 | pMEL1 with 500 bp arms from RK2 (coordinates 38,075-38,573: arm 1; 39,555-40,056: arm 2) to insert antiF cassette; Cam^R^ | This study |
| pMILL2 | pMILL1 with anti-IncF cassette inserted as a *Bgl*II-*Aat*II fragment; Cam^R^ | This study |
| pLAZ1 | pMILL1 without EcoRI site in *cat*; Cam^R^ | This study |
| pLAZ2 | pLAZ1 with anti-F cassette inserted as a *Bgl*II-*Aat*II fragment; Cam^R^ | This study |
| pLAZ2.1 | pLAZ2 with pEK499 copAB inserted in EcoRI site of anti-F cassette; Cam^R^ | This study |
| pLAZSOE1 | pMEL1 with SOEd arms for pUB307 deletion. Cloned into HindIII and SalI sites; Cam^R^ | This study |
| pLAZSOE4 | pMEL1 with 2x 500 bp from RK2 to introduce the i10 region back into pUB307; Cam^R^ | This study |
| pSLK1 | pMILL1 with anti-IncK cassette inserted as an NcoI-BamHI fragment; Cam^R^ | This study |
| RK2Δa*ph* | IncP-1α, Amp^R^, Tet^R^ , Δ*aph* from RK2 mediated by pMILL1 | This study |
| RK2Δ307 | IncP-1α, Kan^R^ Tet^R^, RK2 with site directed deletion identical to that in pUB307 made using pLAZSOE1 | This study: Fig. 3 |
| RK2Δ*klcA-korC* | IncP-1α, Kan^R^ Tet^R^ spontaneous delet^n^ between *klcAp* & *kleAp* (bases 4340-11669 in RK2 map) | This study; Fig. 3 |
| pUB307Δ*aph* | IncP-1α, Tet^R^, Δ*aph* from pUB307 mediated by pMILL1 | This study |
| pUB307::iteron10 | pUB307 with iteron 10 after recombineering with pLAZSOE4 | This study; Fig. 3 |
| pCURE-F-RK2 | IncP-1α, Amp^R^, Tet^R^, antiF cassette inserted via pMILL2 into RK2 | This study; Fig. 3 |
| pCURE-F-307 | IncP-1α, Tet^R^, antiF cassette inserted via pMILL2 into pUB307 | This study; Fig. 3 |
| pCURE-F-9242 | IncP-1α, Tet^R^, antiF cassette inserted via pMILL2 into pR9242 | This study; Fig. 3 |
| pCURE-F- RK2 Δ*klcA-korC* | IncP-1α, Tet^R^, RK2Δ*klcA-korC*-derivative with antiF cassette inserted by recombineering using pMILL2 | This study |
| pCURE-F-307::i10 | IncP-1α, Tet^R^, pUB307::iteron10 with antiF cassette inserted by recombineering using pMILL2 | This study |
| pCURE-FEK499-307 | IncP-1α, Tet^R^, antiF cassette with extra copA copB segment from pEK499 inserted via pLAZ2.1 into pUB307 | This study |
| pCURE-K-RK2 | IncP-1α, Amp^R^, Tet^R^, antiK cassette inserted via pSLK1 into RK2 | This study |
| pCURE-K-307 | IncP-1α, Tet^R^, antiK cassette inserted via pSLK1 into pUB307 | This study |
| pCT549 | Mini IncP-1α, Kan^R^Tet^R^, *korA*^+^*incC*^+^*korB*^+^*korF*^+^*korG*^+^*kfrA*^+^*B*^+^*C*^-^ | Thomas et al (1984)^2^ |
| pCURE-F-549 | Mini IncP-1α, Tet^R^, antiF, *korA*^+^*incC*^+^*korB*^+^*korF*^+^*korG*^+^*kfrA*^+^*B*^+^*C*^-^ | This study, Fig. 4 |
| pCURE-F-549::i10 | Mini IncP-1α, Tet^R^, antiF, *korA*^+^*incC*^+^*korB*^+^*korF*^+^*korG*^+^*kfrA*^+^*B*^+^*C*^-^ | This study, Fig. 4 |
| pCURE-F-549::i10Δi1 | Mini IncP-1α, Tet^R^, antiF, *korA*^+^*incC*^+^*korB*^+^*korF*^+^*korG*^+^*kfrA*^+^*B*^+^*C*^-^ | This study, Fig. 4 |
| pCURE-F-549 ΔtrbB-korF | Mini IncP-1α, Tet^R^, antiF, *korA*^+^*incC*^+^*korB*^+^ | This study, Fig. 4 |
| pCT549ΔtrbB-korB | Mini IncP-1α, Tet^R^, antiF, *korA*^+^*incC*^+^ | This study, Fig. 4 |
| pCURE-F-549 ΔtrbB-incC::korB | Mini IncP-1α, Tet^R^, antiF, *korA*^+^*inc*^-^*korB*^+^ | This study, Fig. 4 |
| pRK2501 | Mini IncP-1α, Tet^R^, Kan^R^; *korA*^+^*incC*^+^*korB*^-^ | 33 |
| pRK2501::anti-F | Mini IncP-1α, Tet^R^, Kan^R^; *korA*^+^*incC*^+^*korB*^-^ | This study |

^1^Thomas CM. Complementation analysis of replication and maintenance functions of broad host plasmids RK2 and RP1. Plasmid (1981) 5: 277-291.

^2^Thomas CM, Cross MA, Hussain AAK, Smith CA. Analysis of copy number control elements in the region of the vegetative replication origin of the broad host range plasmid RK2. EMBO J (1984) 3: 57-63.
