# Supplementary material for: Potentiation of curing by a broad-host-range self-transmissible vector for displacing resistance plasmids to tackle AMR
Source: PLoS One. 2020 Jan 15;15(1):e0225202. doi: 10.1371/journal.pone.0225202 (PMC6961859; doi:10.1371/journal.pone.0225202)
Supplement: S3 Table — (DOCX) [file pone.0225202.s003.docx]

**S3 Table. Primers designed and used during this study**

| **Primer** | **Base sequence (5′-3′ where not indicated)^a^** | **Template, comments** |
| --- | --- | --- |
| Amplification of RK2 arms to allow integration in place of the *aph* gene | | |
| RK2 Arm 1F | AGGCgtcgac**CAAAGGGTTCGCAGACTGGGG** | RK2 |
| RK2 Arm 1R | gacgtcGCTAACagatctTCCttaattaa**GGCATCCCTGACAGACAACGC** |  |
| RK2 Arm 2F | ttaattaaGGAagatctGTTAGCgacgtc**CAGGGAGGCGTTCAGGACGAC** |  |
| RK2 Arm 2R | CCGCaagctt**CACAGCCGGGGCATCTTTGAG** |  |
| Amplification of anti-IncF and anti-IncK functions | | |
| Anti-IncFF | GTCgacgtcCCCTGTTATCCCTACCCGG | pCURE2 |
| Anti-IncFR | GCGagatctAGGGTAATCCCGGATCTTCG |  |
| Anti-IncK | AGCCATGGCCATAAGGCATTCAGGA | R387 |
| Anti-IncK | GTGGATCCGCAGGCTCTGCTCG |  |
| AL IncK F | ATGGTGACAAAGAGAGTGCAAC | pCT::*aph* |
| AL IncK R | TTACAGCCCTTCGGCGATG |  |
| Amplification of copAB region from pEK499 | |  |
| 499 copAB F | GTCCAATTGGTCGACCGTCACAATTCTCAAGTCGC | pEK499 |
| 499 copAB R | GTCCAATTGCTCGAGGTCACACCATCCTGCACTTAC | pEK499 |
| Creation of deletion from pUB307 in RK2 | | |
| 307Δ Arm1F | cgcgtcgactagccgtagcacgactcgatg | RK2 |
| 307Δ Arm1R | CAATTACGTCTCCCATTACGACCATGCGC | RK2 |
| 307Δ Arm2F | cgtaatgggagacgtaattgagcatttccaggc | RK2 |
| 307Δ Arm2R | CGGAAGCTTGGCGGACGTTGACACTTGA | RK2 |
| Reinsertion of region with iteron 10 into pUB307 | |  |
| +i10 Arm1F | CGCGTCGACCCGCTAGATCGCAAAGGAT | RK2 |
| +i10 Arm1R | GAATCGGGTATCCCATTACGACCATGCGC | RK2 |
| +i10 Arm2F | CGTAATGGGATACCCGATTCTGCGGTTACA | RK2 |
| +i10 Arm2R | TATGCCGCCGGACGTAATTGAGCATTTCCAGG | RK2 |
| Mutate EcoRI in pACYC184 | ATGCTCATCCGGAGTTCCGTATGGCAATGAAAGACG | pACYC184 |
|  | CGTCTTTCATTGCCATACGGAACTCCGGATGAGCAT | pACYC184 |
| Manipulation of mini-RK2 plasmid pCT549 | | |
| oriV + i10F | ATCgaattccggccgtacccgattc | RK2 |
| oriV+i1R | gagatagatctagcgtggactcaag | RK2 |
| oriV – i10F | CATgaattcgtttagagcgagccaggaaag | RK2 |
| oriV-i1R | TGAagatctaccgcagggaaattctcgtc | RK2 |
| BglII-PacI-HindIII linker | 5’ agcttACGttaattaaATGTACgacgtcCTAa 3’  3’ATGCAATTAATTTACATGCTGCAGGATTCTAG 5’ | Not applicable |
| MfeIPacIoriV | ATCcaattgGATttaattaaccggccgtacccgattc | With i10 |
| oriVEcoRIXbaI | ATtctagatacgaattcTACctcaaggctctcgcgaatg | With i10 |
| MfeIPacIoriV | ATCcaattgGATttaattaagtttagagcgagccaggaaag | Without i10 |
| oriVEcoRIXbaI | ATtctagatacgaattcTACctcaaggctctcgcgaatg | Without i10 |
| ΔkorF-trbBa^b^ | ATGtctagAACTGTCAAAGCGCACCCG | pCT549 |
| ΔkorF-trbBc^b^ | ATGtctagaCGCTGTCTTTGGGGATCAGC | pCT549 |
| ΔkorB-trbBc^b^ | ATGtctagaCCGCAGTCATTGGGAAATCTC | pCT549 |
| ΔincC-trbBc^b^ | ATGtctagaCCGTGACCAAAGTTTTCATCG | pCT549 |
| korB F | AGTGCATGCgaagatggagatttcccaatg | pCT549 |

1. Restriction sites are shown in red.
2. “c” indicates clockwise on the standard map of RK2 ie running with the coordinates in the RK2 Genbank file. “a” indicates anticlockwise.
