# Supplementary material for: Potentiation of curing by a broad-host-range self-transmissible vector for displacing resistance plasmids to tackle AMR
Source: PLoS One. 2020 Jan 15;15(1):e0225202. doi: 10.1371/journal.pone.0225202 (PMC6961859; doi:10.1371/journal.pone.0225202)
Supplement: S1 Fig — A) Comparison of kanamycin resistance of C600, C600[RK2] and C600[pUB307]. C600[RK2] has an MIC of 600μg ml-1, whereas C600[pUB307] is giving an MIC of 1000–1200μg ml-1. B) Comparison of kanamycin resistance of the same strains as tested in panel A but including additional plasmids. C600[pUB307] is still showing higher resistance than C600[RK2] while C600[pUB307::i10] gives resistance to levels of kanamycin (400–800μg ml-1) more similar to RK2 than to pUB307. C600[RK2𠎔4340–11669] also gives similar resistance to RK2. (DOCX) [file pone.0225202.s004.docx]

**S1 Figure. Growth of *E. coli* C600 with different plasmids at increasing concentrations of kanamycin.** A) Comparison of kanamycin resistance of C600, C600[RK2] and C600[pUB307]. C600[RK2] has an MIC of 600µg ml^-1^, whereas C600[pUB307] is giving an MIC of 1000-1200µg ml^-1^. B) Comparison of kanamycin resistance of the same strains as tested in panel A but including additional plasmids. C600[pUB307] is still showing higher resistance than C600[RK2] while C600[pUB307::i10] gives resistance to levels of kanamycin (400-800µg ml^-1^) more similar to RK2 than to pUB307. C600[RK2∆4340-11669] also gives similar resistance to RK2.
