# Supplementary material for: Potentiation of curing by a broad-host-range self-transmissible vector for displacing resistance plasmids to tackle AMR
Source: PLoS One. 2020 Jan 15;15(1):e0225202. doi: 10.1371/journal.pone.0225202 (PMC6961859; doi:10.1371/journal.pone.0225202)
Supplement: S2 Fig — A. All plasmids compared in triplicate. B. Six isolates pCT549 and pCT549+i10 compared in pairs, overexposed to make the RK2-derived plasmid bands more visible. Band intensities were determined using Quantity One software after adjusting the exposure to ensure that the image was not saturated and then normalised by taking the ratio to the control pDS3 band–numerical data is shown in S1 Table. To give maximum confidence in the pCT549 v pCT549+i10 comparison these two plasmids were also compared alone with multiple samples paired to ensure identical conditions. S1 Table shows the signal for each band after subtracting background. Means and standard deviations were calculated and statistical analysis performed for the critical pCT549 v pCT549+i10 comparison. S1 Raw Images shows the uncropped images used in this Figure. (DOCX) [file pone.0225202.s005.docx]

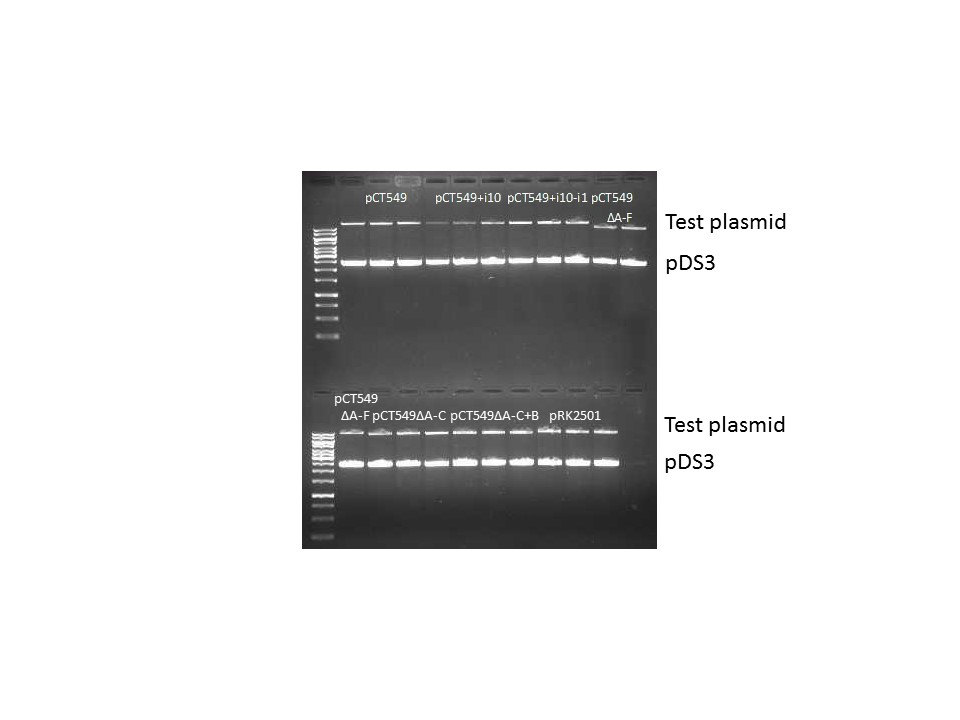
A


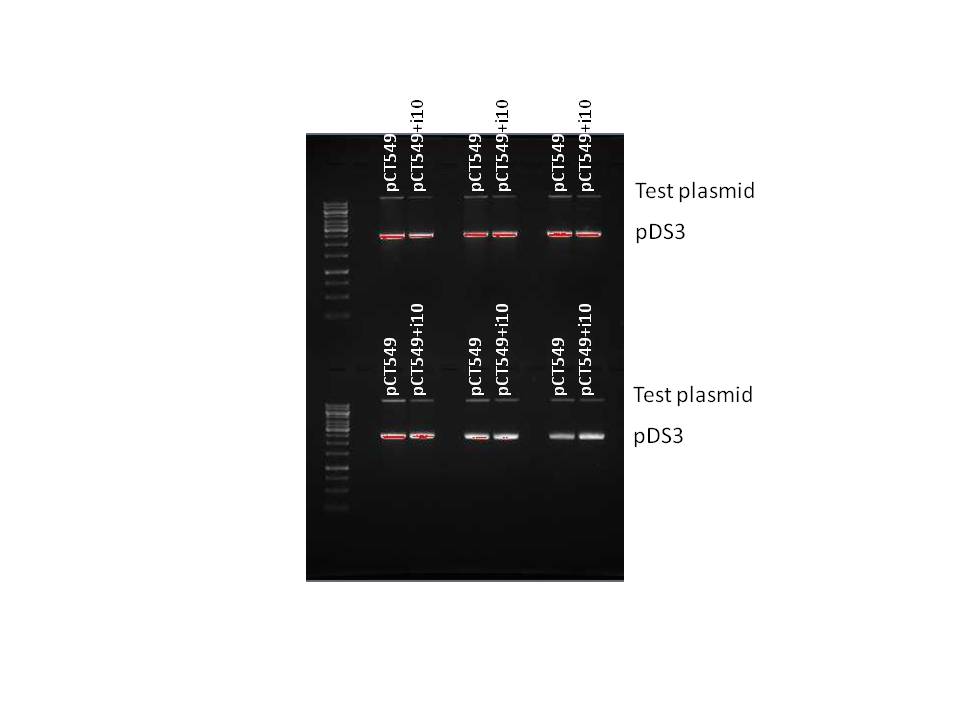


B

**S2 Figure. Comparison of plasmid copy number for key plasmids after curing with EcoRI.** A. All plasmids compared in triplicate. B. Six isolates pCT549 and pCT549+i10 compared in pairs, overexposed to make the RK2-derived plasmid bands more visible. Band intensities were determined using Quantity One software after adjusting the exposure to ensure that the image was not saturated and then normalised by taking the ratio to the control pDS3 band – numerical data is shown in Table in S1_Table. To give maximum confidence in the pCT549 v pCT549+i10 comparison these two plasmids were also compared alone with multiple samples paired to ensure identical conditions. The Table in S1_Table shows the signal for each band after subtracting background. Means and standard deviations were calculated and statistical analysis performed for the critical pCT549 v pCT549+i10 comparison.
