# Supplementary material for: Potentiation of curing by a broad-host-range self-transmissible vector for displacing resistance plasmids to tackle AMR
Source: PLoS One. 2020 Jan 15;15(1):e0225202. doi: 10.1371/journal.pone.0225202 (PMC6961859; doi:10.1371/journal.pone.0225202)
Supplement: S3 Fig — Both HB101 and MV10nalR were successfully used as E. coli donor host strains. Transfer into the donor in the experiment with MV10nalR as the host was detected by selecting resistance to nalidixic acid for the host and kanamycin for the plasmid. Mating mixtures were re-suspended in saline and after serial dilution 20 μl aliquots were spotted in a circle round the plate. The numbers are low so it was not easy to plot them on the same log scale as the data shown in Fig 5. (DOCX) [file pone.0225202.s006.docx]

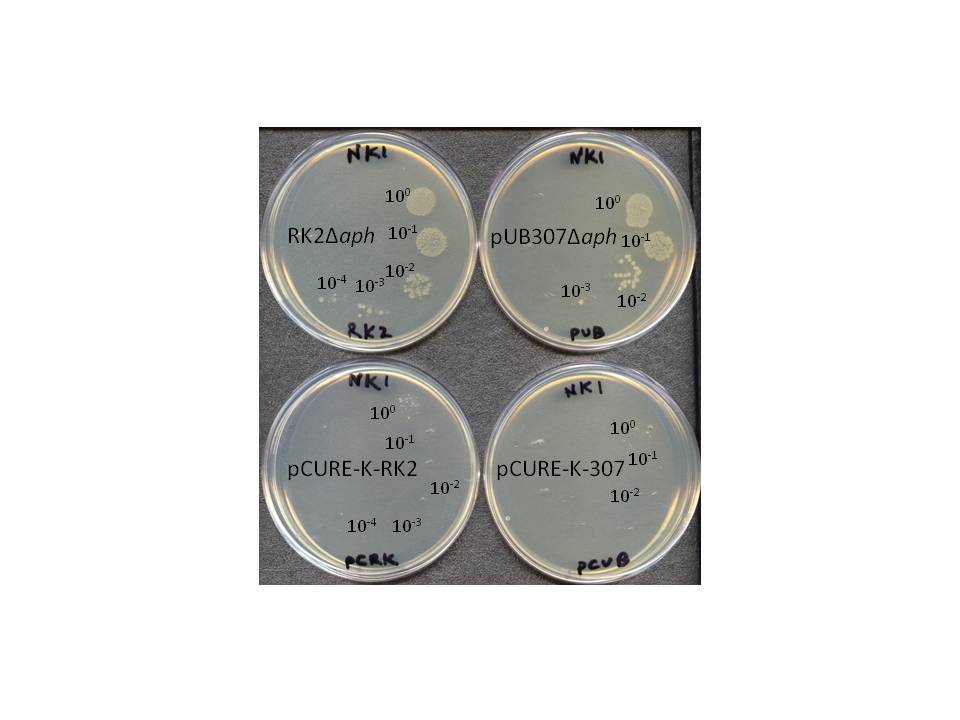


**S3 Figure. Invasion of the donor strain in the unselected invasion assay with pCT::*aph* as the target plasmid.** Both HB101 and MV10nal^R^ were successfully used as *E. coli* donor host strains. Transfer into the donor in the experiment with MV10nal^R^ as the host was detected by selecting resistance to nalidixic acid for the host and kanamycin for the plasmid. Mating mixtures were re-suspended in saline and after serial dilution 20 µl aliquots were spotted in a circle round the plate. The numbers are low so it was not easy to plot them on the same log scale as the data shown in Figure 5.
