# Supplementary material for: Potentiation of curing by a broad-host-range self-transmissible vector for displacing resistance plasmids to tackle AMR
Source: PLoS One. 2020 Jan 15;15(1):e0225202. doi: 10.1371/journal.pone.0225202 (PMC6961859; doi:10.1371/journal.pone.0225202)
Supplement: S4 Fig — The key points are that the frequency of RifR E. coli (pCURE-K-307) is very low and that pCT::aph persists throughout the experiment. The data is shown in the Tables in S4 text. (DOCX) [file pone.0225202.s007.docx]

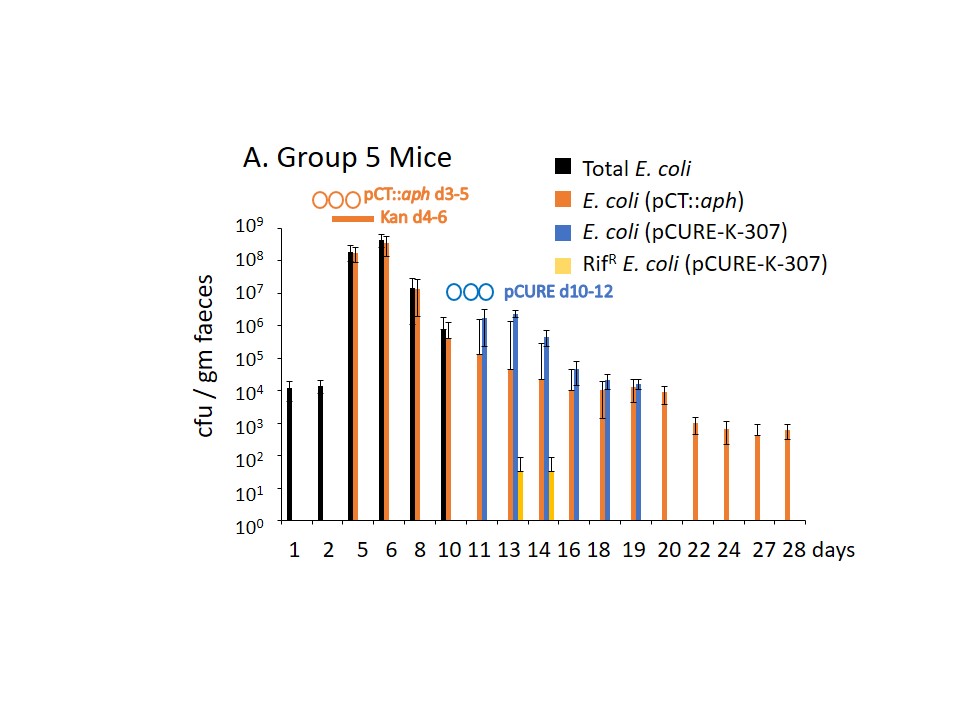


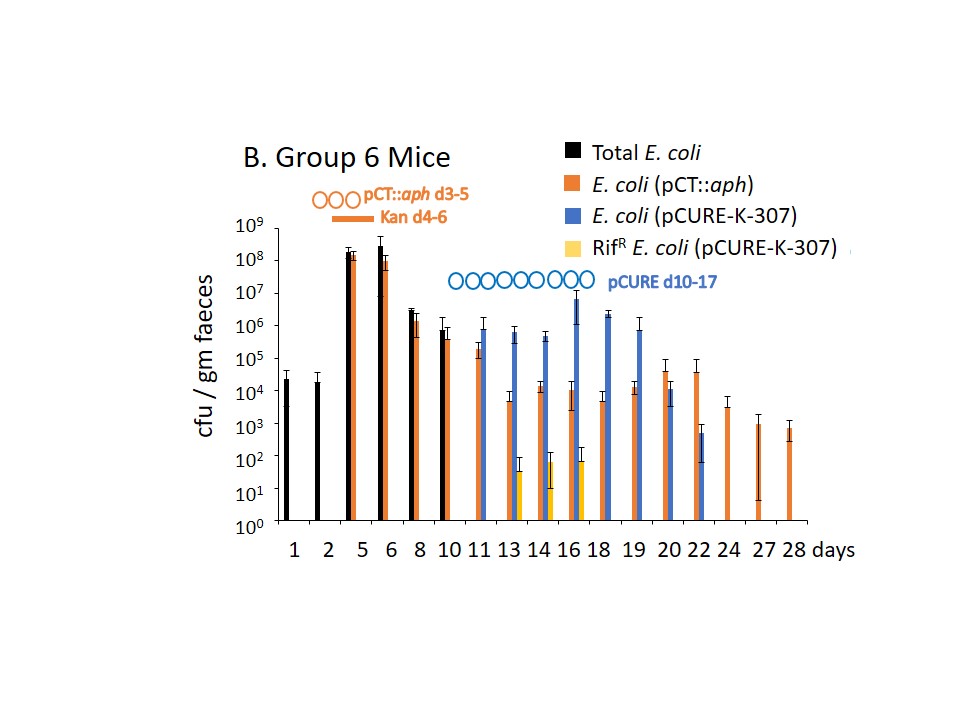


**S4 Figure. Effect of pCURE-K-307on carriage of pCT::*aph* in the absence of selection with tetracycline for comparison with the data in Figure 6C and S6 Figure.** The key points are that the frequency of Rif^R^ *E. coli* (pCURE-K-307) is very low and that pCT::*aph* persists throughout the experiment.
