# Supplementary material for: Potentiation of curing by a broad-host-range self-transmissible vector for displacing resistance plasmids to tackle AMR
Source: PLoS One. 2020 Jan 15;15(1):e0225202. doi: 10.1371/journal.pone.0225202 (PMC6961859; doi:10.1371/journal.pone.0225202)
Supplement: S5 Fig — In the mouse experiments three mice were used for each treatment and the mean at each time point plotted but the data in this Figure is for each individual mouse that was used to generate Fig 6C. The data is shown in the Tables in S4 text. (DOCX) [file pone.0225202.s008.docx]

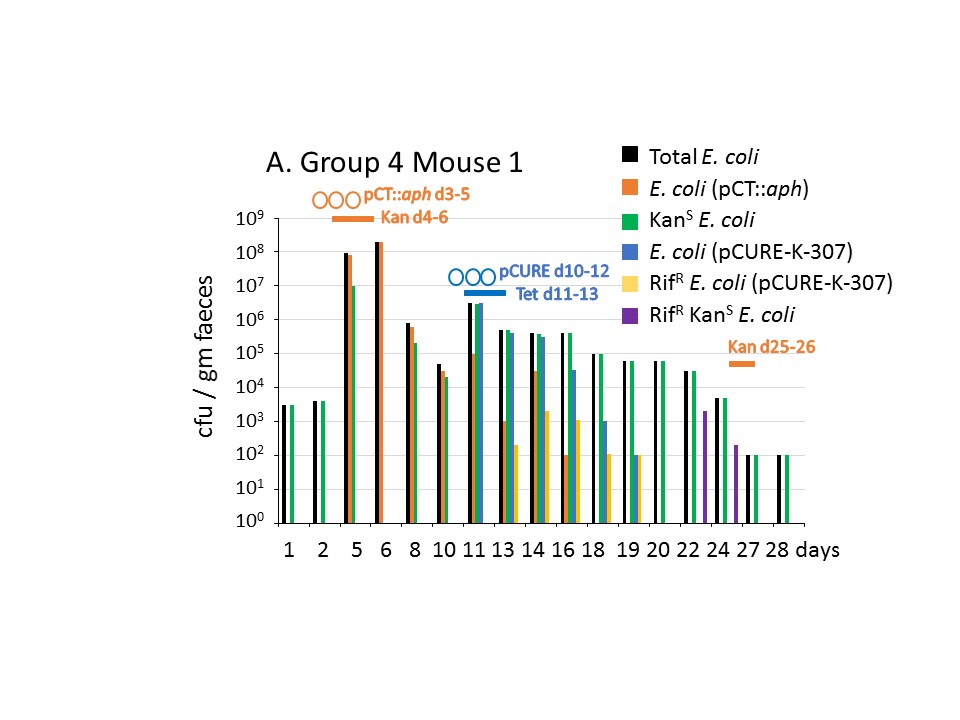


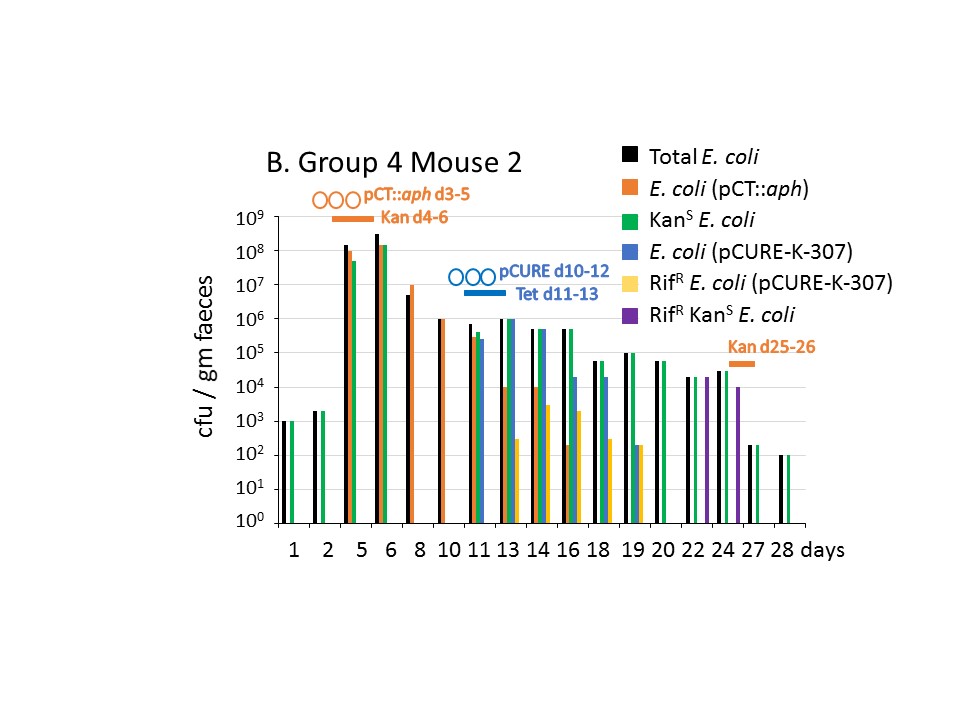


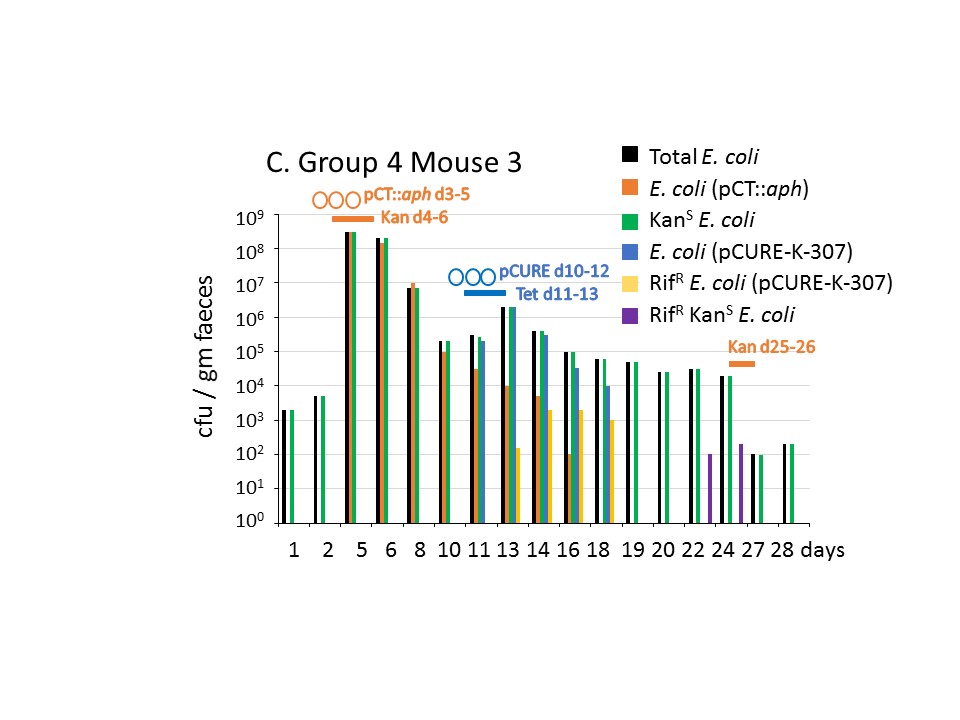


**S5 Figure. Effect of pCURE-K-307 on presence of target plasmid pCT::*aph* in bacteria in the mouse gut when accompanied with a short period of tetracycline treatment after pCURE-K-307 was administered.** In the mouse experiments three mice were used for each treatment and the mean at each time point plotted but the data in this Figure is for each individual mouse that was used to generate Figure 6C.
