# Supplementary material for: Potentiation of curing by a broad-host-range self-transmissible vector for displacing resistance plasmids to tackle AMR
Source: PLoS One. 2020 Jan 15;15(1):e0225202. doi: 10.1371/journal.pone.0225202 (PMC6961859; doi:10.1371/journal.pone.0225202)
Supplement: S6 Fig — The initial alignment was constructed in Clustal Omega but then optimised manually since the sequence divergence, particularly for pQKH54, makes some of the initial alignments unreliable. The Iteron consensus for pQKH54 is also slightly different from that of the other plasmids. Plasmid pRWC72a was chosen as the IncP-1β plasmid because it is like the other plasmids chosen in not having addition system genes between the klcA promoter and Iteron 10. The numbers at the right hand end of the lines refer to the coordinates in the Genbank files for these plasmids. The sequence is not extended as far as Iteron 1 because the size of the Figure then becomes too cumbersome. (DOCX) [file pone.0225202.s009.docx]

Iteron 11

pQKH54 cagtaggcgtgtagcgcctctttcagtcgttgacacttgagggatagtg 8910

Iteron 10

pQKH54 tttgcaggacgtggcggctcttcaaaaccatgacacttgagggatttcacaaaacctggc 8970

pEST4011 ctattccgtcaacccgtacccgcgtttctttgacacttgaggggccacttct-------- 8817

RK2 cgttgaatggtgatcgccatcacgtttcattgacacttgaggggcgttta---------- 11883

pRWC72a cccttgggacgactgaatttcctggcttcttgacacttgaggggcgcatt---------- 51154

R751 gcttcttgacacttgaggggcgcaat---------- 9215

pKJK5 -----tttagccgctaaaatcttgtcatcttgacacttgaggggcgcttt---------- 142

***************************************************************

pQKH54 tttacctgttgacacttaccgttagctaagcggcgcagt 9009

pQKH54 acccccggatggccgcatagcccgccg-ccgacacccctctggcgtaaaa--ccttgcgtt 9068

pEST4011 cccagcaatccggctaacggtcgccggcccttttccccttggagtaaaa--ccttgcgtc 8875

RK2 gagcgagccaggaa-------agccgaccccct---ccttggagtaaaaacccttgcggc 11933

pRWC72a ---cacgcccctggcctgccaggccgaccccctt--ccttggcgttcaa--ccttgcggc 51207

R751 ---cacgccctgggcctggcaggccgacccctc---ccttggcgttcaa--ccttgcggc 9267

pKJK5 --------ccctggccgcgtcggccgaccttcttt-ccttggagtaaaa--cttttcggc 191

**************************************************************************

pQKH54 ctttcatacggcgctgtcgttcttgctgcgtagcagccc----ccgcaggccgcttttca 9123

pEST4011 cgttgcgct-gcgcggtccttcc-atcgtggccgcgatgg-----ttgagctgcatggag 8928

RK2 gttgcagccggcacggatcttccgatcgggcgcggtggtggccgcgtctgtgacctaaa- 11992

pRWC72a cttcgagccggcgctgtcgtcc-gtcagggccacgacgctgaagtcaccgcggccttgac 51266

R751 cttcgagctggagccatcttcc-gacttggcgacggcgcaggagtcaccgcggccttgac 9326

pKJK5 cttccagcttgcgctgatcttcgattagtgcgcgaacggtggtcacgctgcggcctggac 251

**************************************************************************

pQKH54 ggcgcttgcgcctgtttcaccaagc-aagctatgcctttgacgttgctttgctgtc---- 9178

pEST4011 ---agttgcaggagtccagaggggc---gcagcccctttgggcatagcgcagcgacctag 8982

RK2 ----aaggggggagtccagaggggc---gcagcccctttgggcatagcgcagcgtaatcg 12045

pRWC72a ----agttggggagtccagagggggccttggccccctttggaagagc------------- 51309

R751 ----agttggggagtccagagggggccttggccccctttggaagagc------------- 9369

pKJK5 ----agtcggggagtccagaggggc--ggtagcccctttgggcatagcgcagcgtaagc- 304

****************************************************************

pQKH54 --------ctggagcctctgcgcgggctgtagacctgcctggggtgaggccgcaggccga 9230

pEST4011 agacgaaattaaggctgttgca-ggcgcttgcgcctgtttggtagccctctgcgc-cgtt 9040

RK2 gagacgtaattgagcatttcca-ggcgcttgcgcctggtcaacgaaagagtcagcgccgt 12104

pRWC72a --------acgaacttctgcca-ggcgcttgcgcctggctatggtcagccgtagcgccgt 51360

R751 --------acgaacttctgcca-ggcgcttgcgcctg-ctatggtcagccgtagcgccgt 9419

pKJK5 --gcacaaaatttgcttctcca-ggcgcttgcgcctggttc-aagggagcgcagcgccgt 360

**************************************************************************

pQKH54 ggggcttgcccctccaggcgcaagcgcctggtcaacaaggcaaattttgctcttcggggg 9290

pEST4011 -aggcgcagctcgaatgcaggtgaggccgaa---ggccgaggggcgcagcccctgggggg 9095

RK2 -aggcgctgccatttttggggtgaggccgttcgcggccgaggggcgcagcccctgggggg 12163

pRWC72a -aggcgct-ccattcttggggtgaaggccgca--ggccgaggggcaacgcccctgggggg 51416

R751 -aggcgct-ccattcttggggtgaaggccgta--ggccgaggggcaacgcccctgggggg 9475

pKJK5 -aggcgctccatttttggggtgaggacgcgcagcggccgaggggc-ctgcccct-ggggg 415

**************************************************************************

pQKH54 gatgggagaggcgcgcagcgactcgggtgggg-tcgagaaggggggtgtccccccttcggc 9350

pEST4011 atgggaggcc--gctt-gcgg-ccgggt-gggatcgagaaggggggtctccccccttcggc 9152

RK2 atgggaggcccgcgttagcgggccggg-agggttcgagaagggggggcaccccccttcggc 12223

pRWC72a atgggaggcc-gctttagcgg-ccgggt-gggatcgagaagggggggcaccccccttcggc 51474

R751 atgggaggcc-gctttagcgg-ccgggtggggatcgagaagggggggcaccccccttcggc 9534

pKJK5 atgggaggcc-gcgc-agcgg-ccgggt-gggttcgagaagggggg-ca-cccccttcggc 472

***************************************************************************

pQKH54 -gtgccctg----ctgcgcggccgcttt--cgtctttgcgcgttttttaagcgatgtggttat 9406

pEST4011 -------------------------------------gcgtgctttttaagcacgcaggtt- 9176

RK2 -------------gtgcgcggtcacg--------------cgccagggcgcagccctggt-- 12256

pRWC72a ggccggtcatt--ctgcgcggcaccttgggccggtttgccgctttttttcgacacccggtt- 51533

R751 ggccgatcatt--ctgcggggcaccttgggccggtttgccgctttttt-cgacacccggtt- 9592

pKJK5 ggccgattttcgcgccgcgcctggttttttccggcttggcagttttttcgccagcctggt-- 533

**************************************************************************

pQKH54 aaaaaagatggttattgttttaatggttataaaagcggttaaaagaagggttaggcgac- 9465

pEST4011 -aataactacggtttataaaatttggtttaaaaagtggttataaagc-ggttagcgcat- 9233

RK2 -taaaaacaaggtttataaatattggtttaaaagcaggttaaaagacaggttagcggtgg 12315

pRWC72a -ataactaccaggtttaagataacggtta--tgaagggttaaaagcc-ggttagcttga- 51588

R751 -ataacaactaggtttaatataaaggtta--tgaagggttaaaagcc-ggttagcttga- 9647

pKJK5 -taaagaacctagttatatataaaggttataaaagcggttaaaaacctggttagaggct- 590

* * **** ***** ** ******

Iteron 9

pQKH54 ctcgaaaaccgctgtaaagccttgatacgccaagggaaagcacctgctggcaatccccca 9526

pEST4011 cggaaaacatgccgtaagtcgttgatacgccaagaaaaaagc-aaaaccgctgcccctca 9292

RK2 ccgaaaaacgggcggaaacccttgcaaatgctggattttctgcctgtggacagcccctca 12382

pRWC72a ccggaaaacgcgcgcaacccgttgatacgccaaggaaaaggctagaagggccgcccctca 51648

R751 ccggaaaacgcgcgcaagccgttgatacgccaaggaaaaggctcgaagggccgcccctca 9707

pKJK5 ctgaaaaacgcctgtaagccgttgatactccaagggaaaggccgaaggggccgcccctca 650

* *** * ** * *** * * * * *** **

Iteron 8 Iteron 7

pQKH54 agggtcaa-gaaagcatccctcaagggtctatagcctatccctcaagggtcaaaga-aac 9583

pEST4011 agtgtcta-gggtgcgcccctcaaatgtctgtagttggccccacaagtgtcaacgaagcc 9351

RK2 aatgtcaataggtgcgcccctcatctgtcatcactctgcccctcaagtgtcaaggatcgc 12435

pRWC72a agtgtcaatggataagcccctcaagggtcaatagtctgcccgccaagtgtcaaggttcgc 51708

R751 agtgtcaatggataagcccctcaagggtcaatagtctgcccgtcaagtgtcaaggatcgc 9767

pKJK5 agtgtcaatggataagccccccaagtgtcagcagccggcccctcaagtgttaggaaagcc 710

* *** ** *** * ** **** ** * *

Iteron 6 Iteron 5

pQKH54 gtccctcaagggtcaatagccctatccctcaagggtcaataggtagcagcctttatgcac 9643

pEST4011 gccccccaagtgtctagggttgcgcccctcaagtgtctatagaaatcgggcttt-tccac 9410

RK2 gcccctcatctgtcagtagtcgcgcccctcaagtgtcaataccgcagggcacttatcccc 12495

pRWC72a gcccctcatctgtcagcacccccgcccctcaagtgtcagcacggtaggggccttgccaac 51768

R751 gcccctcatctgtcagcacccccgcccctcaagtgtcagtacggcaagggccttgccaac 9827

pKJK5 gcccctcaagtgttagtagctgcgcccctcaagtgtcagtagggcagggcacttgccaac 770

* *** ** ** ******** *** * * ** *

pQKH54 agggttatccacagaaactggggataacatccaccagggccgctttccgcatg------- 9696

pEST4011 aggcttatgcacagttcctgtgcataacttgaccgcggcgagcgc----------ttgca 9460

RK2 aggcttgtccacatcatctgtgggaaactcgcgtaaaatcaggcgttttcgccgatttgc 12556

pRWC72a aggcttatccacaacttctgtggataaagcctgtcggatcaagcgcataggccgcctcga 51828

R751 aggcttatccacaacttctgtggataaagccagcccgatcaagcacataggccgcctcga 9887

pKJK5 agggttatccacaattcctgtggaaaactcccgcgaaatcagccggatagagtccctgga 830

*** ** * **** *** * **

Iteron 4

pQKH54 ----ttccgtacccggtggttatgccctgaaatgtcac---tatcccccatgtgtcagtacc 9751

pEST4011 --ggcacttggccacgcctggctaggctgaaaaccgacgtttgcccctcaagtgtctgcagt 9520

RK2 gaggctggccagctccacgtcgccggccgaaatcgag--cctgcccctcatctgtcaacgcc 12615

pRWC72a -cggccgccggcgcagctccgggcggccgatttcgag-gccagcccctcatctgtcagcatc 51888

R751 -cggcctccggcgcggctccagccggccgatttcgag-gccggcccctcatctgtcagcatc 9947

pKJK5 --acgcgctcgcgggcctcgatctggccgaaatcgg-ccggtgcccctcatgtgtcagcagt 889

***************************************************************************

Iteron 3 Iteron 2

pQKH54 gtgccgggggtagttatccctcatgtgtcaaccaaacacgtcttcactgcttcacacctg 9811

pEST4011 gcgccggggtactctgccccccaagtgtcaatgaacgccccccaagtgtcacggtgagcc 9580

RK2 gcgccgggtgagtcggcccctcaagtgtcaacgtccgcccctcatctgtcagtgagggcc 12675

pRWC72a gcgccggggtgcgctgccccccaagtgtcaagaaacgcccctcaactgtcaccggccggc 51948

R751 gcgccggggtgcgctgccccccaagtgtcaagaaacgcccctcaactgtcaccagccggc 10007

pKJK5 gcgccggcatggtcggcccctcaagtgttaacaagcgcccttcaagggtcatcgtcggcc 948

***************************************************************************

**S6 Fig. Alignment of the *oriV* regions of plasmids representing IncP-1 subgroups α (RK2, BN000925.1), β (pRWC72a, JX486125.1; R751, NC_001735.4), γ (pQKH54, AM157767.1), δ (pEST4011, NC_005793.2) and ε (pKJK5, AM261282.1) to show the conservation of Iteron 10 in all five groups.** The initial alignment was constructed in Clustal Omega but then optimised manually since the sequence divergence, particularly for pQKH54, makes some of the initial alignments unreliable. The Iteron consensus for pQKH54 is also slightly different from that of the other plasmids. Plasmid pRWC72a was chosen as an IncP-1β plasmid because it is like the other plasmids chosen in not having additional system genes between the *klcA* promoter and Iteron 10. The numbers at the right hand end of the lines refer to the coordinates in the Genbank files for these plasmids. The sequence is not extended as far as Iteron 1 because the size of the Figure then becomes too cumbersome.
