# Supplementary material for: Potentiation of curing by a broad-host-range self-transmissible vector for displacing resistance plasmids to tackle AMR
Source: PLoS One. 2020 Jan 15;15(1):e0225202. doi: 10.1371/journal.pone.0225202 (PMC6961859; doi:10.1371/journal.pone.0225202)
Supplement: S2 Text — (DOCX) [file pone.0225202.s011.docx]

**S2 Text. Data for displacement of pCT::*aph* shown in Fig 4B.**

Numbers are cfu ml^-1^ bacterial suspension from the mating filters in trplicate on days 0, 1, 3, 5 or days 0, 1, 2, 3, 4, 5.

RK2Δ*aph*

| Day | HB101[RK2Δaph] | | | mean | SD |
| --- | --- | --- | --- | --- | --- |
| 0 | 7.2E+05 | 6.0E+05 | 1.3E+06 | 8.9E+05 | 3.9E+05 |
| 1 | 1.3E+06 | 1.8E+07 | 2.0E+07 | 1.3E+07 | 1.0E+07 |
| 3 | 3.9E+07 | 4.1E+07 | 2.9E+08 | 1.3E+08 | 1.5E+08 |
| 5 | 1.3E+08 | 5.6E+07 | 1.8E+07 | 6.8E+07 | 5.6E+07 |

| Day | J53rif total | | | mean | SD |
| --- | --- | --- | --- | --- | --- |
| 0 | 1.3E+09 | 1.6E+09 | 1.5E+09 | 1.5E+09 | 1.4E+08 |
| 1 | 4.3E+09 | 4.6E+09 | 2.7E+09 | 3.9E+09 | 9.9E+08 |
| 3 | 2.8E+09 | 8.1E+08 | 1.5E+09 | 1.7E+09 | 1.0E+09 |
| 5 | 2.4E+09 | 2.6E+09 | 2.4E+09 | 2.5E+09 | 1.3E+08 |

| Day | J53rif(pCT::aph) | | | mean | SD |
| --- | --- | --- | --- | --- | --- |
| 0 | 1.6E+09 | 1.5E+09 | 1.3E+09 | 1.5E+09 | 1.8E+08 |
| 1 | 4.5E+09 | 4.4E+09 | 3.2E+09 | 4.0E+09 | 7.1E+08 |
| 3 | 2.8E+09 | 1.2E+09 | 2.1E+09 | 2.1E+09 | 8.1E+08 |
| 5 | 2.4E+09 | 2.9E+09 | 2.2E+09 | 2.5E+09 | 3.5E+08 |

| Day | J53rif[RK2Δaph] | | | mean | SD |
| --- | --- | --- | --- | --- | --- |
| 0 | 0 | 0 | 0 | 0 | 0 |
| 1 | 6.6E+07 | 2.9E+08 | 1.1E+08 | 1.6E+08 | 1.2E+08 |
| 3 | 2.2E+09 | 1.2E+09 | 1.7E+09 | 1.7E+09 | 5.2E+08 |
| 5 | 7.0E+08 | 2.6E+09 | 2.0E+09 | 1.8E+09 | 9.8E+08 |

pCURE-K-RK2

| Day | HB101[pCURE-K-RK2] | | | mean | SD |
| --- | --- | --- | --- | --- | --- |
| 0 | 1.1E+06 | 4.5E+05 | 8.4E+05 | 8.0E+05 | 3.3E+05 |
| 1 | 1.8E+07 | 9.6E+06 | 1.7E+07 | 1.5E+07 | 4.4E+06 |
| 3 | 2.3E+07 | 3.8E+07 | 6.5E+07 | 4.2E+07 | 2.1E+07 |
| 5 | 2.6E+08 | 1.2E+08 | 2.3E+08 | 2.0E+08 | 7.0E+07 |

| Day | J53rif total | | | mean | SD |
| --- | --- | --- | --- | --- | --- |
| 0 | 1.7E+09 | 1.7E+09 | 1.9E+09 | 1.8E+09 | 1.1E+08 |
| 1 | 4.6E+09 | 4.2E+09 | 4.5E+09 | 4.5E+09 | 1.9E+08 |
| 3 | 2.8E+09 | 2.7E+09 | 3.1E+09 | 2.9E+09 | 1.8E+08 |
| 5 | 2.7E+09 | 1.7E+09 | 2.1E+09 | 2.2E+09 | 4.7E+08 |

| Day | J53rif[pCT::aph] | | | mean | SD |
| --- | --- | --- | --- | --- | --- |
| 0 | 1.8E+09 | 1.2E+08 | 1.9E+09 | 1.3E+09 | 989283268 |
| 1 | 4.4E+09 | 4.0E+09 | 4.2E+09 | 4.2E+09 | 181012426 |
| 3 | 2.6E+09 | 2.9E+09 | 2.6E+09 | 2.7E+09 | 186532985 |
| 5 | 2.9E+09 | 1.8E+09 | 3.0E+09 | 2.6E+09 | 671715678 |

| Day | J53rif[ [pCURE-K-RK2] | | | mean | SD |
| --- | --- | --- | --- | --- | --- |
| 0 | 0 | 0 | 0 | 0 | 0 |
| 1 | 2.9E+08 | 9.9E+07 | 3.0E+08 | 2.3E+08 | 1.1E+08 |
| 3 | 2.0E+09 | 2.3E+09 | 1.7E+09 | 2.0E+09 | 2.8E+08 |
| 5 | 1.3E+09 | 8.4E+08 | 1.8E+09 | 1.3E+09 | 4.7E+08 |

pUB307Δ*aph*

| Day | HB101[pUB307Δ*aph*] | | | mean | SD |
| --- | --- | --- | --- | --- | --- |
| 0 | 1.1E+06 | 1.1E+06 | 3.2E+05 | 8.1E+05 | 4.3E+05 |
| 1 | 1.3E+07 | 7.2E+06 | 2.9E+06 | 7.6E+06 | 4.9E+06 |
| 2 | 2.1E+08 | 2.2E+08 | 1.8E+08 | 2.0E+08 | 2.2E+07 |
| 3 | 1.6E+08 | 3.8E+07 | 1.1E+08 | 1.0E+08 | 6.2E+07 |
| 4 | 3.2E+08 | 2.5E+08 | 2.6E+08 | 2.8E+08 | 3.7E+07 |
| 5 | 8.3E+07 | 1.8E+08 | 2.1E+08 | 1.6E+08 | 6.7E+07 |

| Day | J53Rif total | | | mean | SD |
| --- | --- | --- | --- | --- | --- |
| 0 | 1.4E+09 | 1.4E+09 | 1.8E+09 | 1.5E+09 | 2.3E+08 |
| 1 | 2.0E+09 | 3.0E+09 | 1.2E+09 | 2.1E+09 | 8.7E+08 |
| 2 | 4.8E+09 | 5.2E+09 | 4.3E+09 | 4.8E+09 | 4.2E+08 |
| 3 | 2.1E+09 | 2.0E+09 | 1.7E+09 | 1.9E+09 | 2.5E+08 |
| 4 | 3.0E+09 | 2.2E+09 | 2.7E+09 | 2.6E+09 | 4.1E+08 |
| 5 | 5.8E+08 | 2.2E+09 | 2.2E+09 | 1.7E+09 | 9.5E+08 |

| Day | J53Rif[pCT::aph] | | | mean | SD |
| --- | --- | --- | --- | --- | --- |
| 0 | 1.6E+09 | 1.3E+09 | 1.7E+09 | 1.5E+09 | 1.9E+08 |
| 1 | 2.6E+09 | 3.0E+09 | 1.2E+09 | 2.2E+09 | 9.4E+08 |
| 2 | 4.7E+09 | 4.8E+09 | 9.3E+08 | 3.5E+09 | 2.2E+09 |
| 3 | 2.4E+09 | 2.5E+09 | 1.9E+09 | 2.3E+09 | 3.1E+08 |
| 4 | 2.8E+09 | 0.0E+00 | 2.7E+09 | 1.8E+09 | 1.6E+09 |
| 5 | 2.4E+09 | 0.0E+00 | 2.3E+09 | 1.5E+09 | 1.3E+09 |

| Day | J53Rif[pUB307Δ*aph*] | | | mean | SD |
| --- | --- | --- | --- | --- | --- |
| 0 | 0 | 0 | 0 | 0 | 0 |
| 1 | 2.6E+09 | 3.0E+09 | 1.2E+09 | 2.2E+09 | 9.4E+08 |
| 2 | 4.7E+09 | 4.8E+09 | 9.3E+08 | 3.5E+09 | 2.2E+09 |
| 3 | 2.4E+09 | 2.5E+09 | 1.9E+09 | 2.3E+09 | 3.1E+08 |
| 4 | 2.8E+09 | 0.0E+00 | 2.7E+09 | 1.8E+09 | 1.6E+09 |
| 5 | 2.4E+09 | 0.0E+00 | 2.3E+09 | 1.5E+09 | 1.3E+09 |

pCURE-K-307

| Day | HB101[pCURE-K-307] | | | mean | SD |
| --- | --- | --- | --- | --- | --- |
| 0 | 8.1E+05 | 9.3E+05 | 6.6E+05 | 8.0E+05 | 1.4E+05 |
| 1 | 4.7E+07 | 1.1E+07 | 6.9E+06 | 2.2E+07 | 2.2E+06 |
| 2 | 8.0E+07 | 1.0E+08 | 1.4E+08 | 1.1E+08 | 3.3E+07 |
| 3 | 9.9E+07 | 6.8E+07 | 9.8E+07 | 8.8E+07 | 1.8E+07 |
| 4 | 1.1E+08 | 1.8E+08 | 1.7E+08 | 1.5E+08 | 4.0E+07 |
| 5 | 2.4E+08 | 1.8E+08 | 2.1E+08 | 2.1E+08 | 2.8E+07 |

| Day | J53Rif total | | | mean | SD |
| --- | --- | --- | --- | --- | --- |
| 0 | 1.6E+09 | 1.3E+09 | 8.0E+08 | 1.2E+09 | 4.2E+08 |
| 1 | 3.4E+09 | 3.5E+09 | 4.3E+09 | 3.7E+09 | 5.0E+08 |
| 2 | 4.9E+09 | 5.0E+09 | 4.1E+09 | 4.7E+09 | 4.9E+08 |
| 3 | 2.8E+09 | 3.3E+09 | 3.1E+09 | 3.1E+09 | 2.6E+08 |
| 4 | 2.5E+09 | 2.2E+09 | 2.4E+09 | 2.4E+09 | 2.0E+08 |
| 5 | 2.3E+09 | 1.6E+09 | 2.7E+09 | 2.2E+09 | 5.2E+08 |

| Day | J53Rif[pCT::aph] | | | mean | SD |
| --- | --- | --- | --- | --- | --- |
| 0 | 1.7E+09 | 1.6E+09 | 1.3E+09 | 1.5E+09 | 2.5E+08 |
| 1 | 3.5E+09 | 3.9E+09 | 3.7E+09 | 3.7E+09 | 1.8E+08 |
| 2 | 2.9E+09 | 1.3E+09 | 9.0E+08 | 1.7E+09 | 1.0E+09 |
| 3 | 2.1E+07 | 3.0E+07 | 1.5E+07 | 2.2E+07 | 7.7E+06 |
| 4 | 0.0E+00 | 6.0E+06 | 7.1E+06 | 4.3E+06 | 3.8E+06 |
| 5 | 8.7E+05 | 9.9E+05 | 5.1E+06 | 2.3E+06 | 2.4E+06 |

| Day | J53Rif[pCURE-K-307] | | | mean | SD |
| --- | --- | --- | --- | --- | --- |
| 0 | 0 | 0 | 0 | 0 | 0 |
| 1 | 5.3E+08 | 1.4E+08 | 1.2E+08 | 2.6E+08 | 2.3E+08 |
| 2 | 1.3E+09 | 2.6E+09 | 2.3E+09 | 2.0E+09 | 7.0E+08 |
| 3 | 1.6E+09 | 2.2E+09 | 2.0E+09 | 1.9E+09 | 3.2E+08 |
| 4 | 1.6E+09 | 2.2E+09 | 2.0E+09 | 1.9E+09 | 3.3E+08 |
| 5 | 1.2E+09 | 9.0E+08 | 1.7E+09 | 1.3E+09 | 4.2E+08 |
