# Supplementary material for: Potentiation of curing by a broad-host-range self-transmissible vector for displacing resistance plasmids to tackle AMR
Source: PLoS One. 2020 Jan 15;15(1):e0225202. doi: 10.1371/journal.pone.0225202 (PMC6961859; doi:10.1371/journal.pone.0225202)
Supplement: S3 Text — (DOCX) [file pone.0225202.s012.docx]

**S3 Text. Data for the mouse experiment shown in Fig 6A.**

Group 1 mice received E. coli with pCT::*aph* and subsequently received Kanamycin antibiotic for 3 days

| Group 1 | Mouse 1 |  | Mouse 2 |  | Mouse 3 |  |
| --- | --- | --- | --- | --- | --- | --- |
|  | Total  *E. coli* | *E. coli* (pCT::*aph*) | Total  *E. coli* | *E. coli* (pCT::*aph*) | Total  E. coli | E. coli (pCT::*aph*) |
| D3 | 1.3E+08 | 1.1E+06 | 8.0E+07 | 1.0E+06 | 5.0E+07 | 3.3 E+06 |
| D4 | 4.0E+07 | 3.0E+07 | 1.2E+08 | 1.0E+08 | 3.0E+07 | 3.0E+07 |
| D6 | 4.5 E+08 | 4.5E+08 | 1.9E+08 | 1.9E+08 | 2.8E+08 | 3.2 E+08 |
| D8 | 1.0E+07 | 1.0E+07 | 7.0E+06 | 6.0 E+06 | 6.0E+06 | 5.0 E+06 |
| D10 | 2.0E+06 | 1.5E+06 | 7.8E+05 | 8.0 E+05 | 6.5E+05 | 6.0 E+05 |
| D14 | 4.0E+05 | 3.0E+05 | 3.5E+05 | 2.0 E+05 | 3.1E+05 | 1.5 E+05 |
| D17 | 1.0E+05 | 1.7E+04 | 1.5E+05 | 1.1 E+05 | 1.7E+05 | 1.0 E+05 |

Group 2 mice received E. coli with pCT::*aph* but no Kanamycin antibiotic

| Group 2 | Mouse 1 |  | Mouse 2 |  | Mouse 3 |  |
| --- | --- | --- | --- | --- | --- | --- |
|  | Total  *E. coli* | *E. coli* (pCT::*aph*) | Total  *E. coli* | *E. coli* (pCT::*aph*) | Total  E. coli | E. coli (pCT::*aph*) |
| D3 | 4.5E+07 | 2.6E+06 | 8.5E+07 | 4.0E+06 | 6.7E+07 | 2.3E+06 |
| D4 | 6.0E+07 | 5.0E+06 | 2.0E+08 | 6.0E+07 | 2.0E+07 | 1.3E+06 |
| D6 | 3.3E+08 | 4.6E+07 | 4.5E+08 | 3.9E+07 | 6.8E+08 | 3.9E+07 |
| D8 | 3.0E+07 | 7.0E+05 | 8.0E+06 | 5.0E+05 | 7.3E+06 | 3.0E+05 |
| D10 | 2.8E+06 | 6.0E+04 | 5.4E+05 | 8.0E+04 | 5.7E+05 | 3.0E+04 |
| D14 | 5.0E+05 | 5.0E+04 | 5.0E+05 | 3.0E+04 | 3.0E+05 | 5.0E+04 |
| D17 | 2.0E+05 | 1.0E+03 | 1.3E+05 | 1.5E+03 | 1.8E+05 | 3.0E+03 |

Proportion of Kan^R^ bacteria sensitive to Rif. The *E. coli* strain with pCT::aph was Rif^R^. The endogenous E. coli were Rif^S^. To check for transfer to the endogenous *E. coli* at days 4, 6 and 8 100 Kan^R^ colonies were replicated onto agar with and without Rif. Kan^R^ Rif^S^ bacteria were checked by PCR to ensure they did have the plasmid.

|  | Group 1 | | | Group 2 | | |
| --- | --- | --- | --- | --- | --- | --- |
|  | Mouse 1 | Mouse 2 | Mouse3 | Mouse 1 | Mouse 2 | Mouse 3 |
|  | Total  *E. coli* | *E. coli* (pCT::aph) | Total  *E. coli* | *E. coli* (pCT::aph) | Total  E. coli | E. coli (pCT::aph) |
| D4 | 7/100 | 8/100 | 15/100 | 0/100 | 0/100 | 0/100 |
| D6 | 8/100 | 10/100 | 12/100 | 0/100 | 0/100 | 0/100 |
| D8 | 5/100 | 10/100 | 11/100 | 0/100 | 0/100 | 0/100 |
