# Supplementary material for: Potentiation of curing by a broad-host-range self-transmissible vector for displacing resistance plasmids to tackle AMR
Source: PLoS One. 2020 Jan 15;15(1):e0225202. doi: 10.1371/journal.pone.0225202 (PMC6961859; doi:10.1371/journal.pone.0225202)
Supplement: S4 Text — (DOCX) [file pone.0225202.s013.docx]

**S4 Text. Data for the mouse experiments shown in Fig 6B and 6C as well as in S4 Fig and S5 Fig.**

The experiments were carried out as described in the Methods section entitled Mouse Experiments. **Group 1** (control group): received normal food and drink plus sucrose water when other groups received it but without bacteria or antibiotics. **Group 2** (antibiotic control group): received normal food and drink plus sucrose water with antibiotics when groups 3 & 4 received antibiotics. Both groups gave similar numbers of bacteria initially but no resistant bacteria. Only data for mice that were fed bacteria carrying the target plasmid is shown. These numbers are cfu ml^-1^ of saline with 100 mg mouse faeces.

| Group 3 | Mouse 1 |  |  |
| --- | --- | --- | --- |
|  | Total  *E. coli* | *E. coli* (pCT::aph) | Kan^S^  E. coli |
| D1 | 2.0E+03 |  | 2.0E+03 |
| D2 | 2.5E+03 |  | 2.5E+03 |
| D5 | 2.6E+08 | 2.0E+08 | 6.0E+07 |
| D6 | 4.0E+08 | 4.0E+08 | 0.0E+00 |
| D8 | 2.0E+08 | 1.5E+08 | 5.0E+07 |
| D10 | 2.0E+06 | 2.0E+06 | 00E+00 |
| D11 | 1.3E+05 | 1.0E+05 | 3.0E+04 |
| D13 | 1.0E+05 | 1.9E+05 | 0.0E+00 |
| D14 | 4.0E+04 | 3.5E+04 | 5.0E+04 |
| D16 | 3.0E+04 | 2.0E+04 | 1.0E+04 |
| D18 | 3.0E+04 | 3.0E+04 | 0.0E+00 |
| D19 | 3.0E+04 | 2.0E+04 | 1.0E+04 |
| D20 | 1.3E+05 | 1.0E+05 | 3.0E+04 |
| D22 | 3.0E+04 | 1.0E+04 | 2.0E+04 |
| D24 | 5.0E+04 | 3.0E+04 | 2.0E+04 |
| D27 | 5.0E+04 | 1.0E+04 | 4.0E+04 |
| D28 | 1.0E+04 | 5.0E+03 | 7.0E+03 |

| **Group 3** | **Mouse 2** |  |  |
| --- | --- | --- | --- |
|  | Total  *E. coli* | *E. coli* (pCT::aph) | Kan^S^  *E. coli* |
| D1 | 1.0E+04 |  | 1.0E+04 |
| D2 | 5.0E+03 |  | 5.0E+03 |
| D5 | 2.0E+08 | 1.5E+08 | 1.0E+08 |
| D6 | 4.0E+08 | 4.0E+08 | 00E+00 |
| D8 | 2.0E+08 | 1.8E+07 | 2.0E+06 |
| D10 | 6.0E+06 | 6.0E+06 | 0.0E+00 |
| D11 | 1.3E+05 | 1.0E+05 | 3.0E+04 |
| D13 | 1.9E+05 | 1.9E+05 | 0.0E+00 |
| D14 | 4.0E+04 | 3.5E+04 | 5.0E+03 |
| D16 | 3.0E+04 | 2.0E+04 | 1.0E+04 |
| D18 | 3.0E+04 | 3.0E+04 | 0.0E+00 |
| D19 | 3.0E+04 | 2.0E+04 | 1.0E+04 |
| D20 | 1.3E+05 | 1.0E+05 | 3.0E+04 |
| D22 | 3.0E+04 | 1.0E+04 | 2.0E+04 |
| D24 | 5.0E+04 | 3.0E+04 | 2.0E+04 |
| D27 | 1.0E+05 | 2.0E+04 | 8.0E+04 |
| D28 | 1.0E+04 | 4.0E+03 | 6.0E+03 |

| **Group 3** | **Mouse 3** |  |  |
| --- | --- | --- | --- |
|  | Total  *E. coli* | *E. coli* (pCT::aph) | Kan^S^  *E. coli* |
| D1 | 2.0E+04 |  | 2.0E+04 |
| D2 | 1.0E+04 |  | 1.0E+04 |
| D5 | 2.2E+08 | 2.0E+08 | 2.0E+07 |
| D6 | 4.0E+08 | 3.0E+08 | 1.0E+08 |
| D8 | 8.0E+07 | 8.0E+07 | 1.0E+00 |
| D10 | 3.0E+05 | 2.0E+05 | 1.0E+05 |
| D11 | 3.0E+05 | 2.0E+05 | 1.0E+05 |
| D13 | 2.6E+05 | 2.0E+05 | 6.0E+04 |
| D14 | 6.0E+04 | 5.0E+04 | 1.0E+04 |
| D16 | 1.0E+05 | 1.5E+04 | 8.5E+04 |
| D18 | 1.0E+05 | 1.0E+04 | 9.0E+04 |
| D19 | 5.0E+04 | 2.0E+04 | 3.0E+04 |
| D20 | 6.0E+04 | 2.0E+04 | 4.0E+04 |
| D22 | 5.0E+04 | 4.0E+03 | 4.6E+04 |
| D24 | 4.0E+04 | 8.0E+03 | 3.2E+04 |
| D27 | 1.0E+04 | 1.0E+03 | 9.0E+03 |
| D28 | 2.0E+04 | 1.0E+03 | 1.9E+04 |

**Group 4**

| **Group 4 Mouse 1** | Total  *E. coli* | *E. coli* (pCT::aph) | Kan^S^  E. coli | *E. coli*  (pC-K-307) | Rif^R^ E. coli (pC-K-307) | RifR Kn^S^  E. coli |
| --- | --- | --- | --- | --- | --- | --- |
| D1 | 3.0E+03 |  | 3.0E+03 |  |  |  |
| D2 | 4.0E+03 |  | 4.0E+03 |  |  |  |
| D5 | 9.0E+07 | 8.0E+07 | 1.0E+07 |  |  |  |
| D6 | 2.0E+08 | 2.0E+08 | 0.0E+00 |  |  |  |
| D8 | 8.0E+05 | 6.0E+05 | 2.0E+05 |  |  |  |
| D10 | 5.0E+04 | 3.0E+04 | 2.0E+04 |  |  |  |
| D11 | 3.0E+06 | 1.0E+05 | 2.9E+06 | 3.0E+06 |  |  |
| D13 | 5.0E+05 | 1.0E+03 | 5.0E+05 | 4.0E+05 | 2.0E+02 |  |
| D14 | 4.0E+05 | 3.0E+04 | 3.7E+05 | 3.0E+05 | 2.0E+03 |  |
| D16 | 4.0E+05 | 1.0E+02 | 4.0E+05 | 3.3E+04 | 1.1E+03 |  |
| D18 | 1.0E+05 | 1.0E+00 | 1.0E+05 | 1.0E+03 | 1.1E+02 |  |
| D19 | 6.0E+04 | 1.0E+00 | 6.0E+04 | 1.0E+02 | 1.0E+02 |  |
| D20 | 6.0E+04 | 1.0E+00 | 6.0E+04 | 1.0E+00 | 1.0E+00 |  |
| D22 | 3.0E+04 | 1.0E+00 | 3.0E+04 | 1.0E+00 | 1.0E+00 | 2.0E+03 |
| D24 | 5.0E+03 | 1.0E+00 | 5.0E+03 | 1.0E+00 | 1.0E+00 | 2.0E+02 |
| D27 | 1.0E+02 | 1.0E+00 | 9.9E+01 | 1.0E+00 | 1.0E+00 |  |
| D28 | 1.0E+02 | 1.0E+00 | 9.9E+01 | 1.0E+00 | 1.0E+00 |  |

| **Group 4 Mouse 2** | Total  *E. coli* | *E. coli* (pCT::aph) | Kan^S^  *E. coli* | *E. coli*  (pC-K-307) | Rif^R^ *E. coli*  (pC-K-307) | Rif^R^ Kn^S^  *E. coli* |
| --- | --- | --- | --- | --- | --- | --- |
| D1 | 1.0E+03 |  | 1.0E+03 |  |  |  |
| D2 | 2.0E+03 |  | 2.0E+03 |  |  |  |
| D5 | 1.5E+08 | 1.0E+08 | 5.0E+07 |  |  |  |
| D6 | 3.0E+08 | 1.5E+08 | 1.5E+08 |  |  |  |
| D8 | 5.0E+06 | 1.0E+07 | -5.0E+06 |  |  |  |
| D10 | 1.0E+06 | 1.0E+06 | 0.0E+00 |  |  |  |
| D11 | 7.0E+05 | 3.0E+05 | 4.0E+05 | 2.5E+05 |  |  |
| D13 | 1.0E+06 | 1.0E+04 | 9.9E+05 | 1.0E+06 | 3.0E+02 |  |
| D14 | 5.0E+05 | 1.0E+04 | 4.9E+05 | 5.0E+05 | 3.0E+03 |  |
| D16 | 5.0E+05 | 2.0E+02 | 5.0E+05 | 2.0E+04 | 2.0E+03 |  |
| D18 | 6.0E+04 | 1.0E+00 | 6.0E+04 | 2.0E+04 | 3.0E+02 |  |
| D19 | 1.0E+05 | 1.0E+00 | 1.0E+05 | 2.0E+02 | 2.0E+02 |  |
| D20 | 6.0E+04 | 1.0E+00 | 6.0E+04 | 1.0E+00 | 1.0E+00 |  |
| D22 | 2.0E+04 | 1.0E+00 | 2.0E+04 | 1.0E+00 | 1.0E+00 | 2.0E+04 |
| D24 | 3.0E+04 | 1.0E+00 | 3.0E+04 | 1.0E+00 | 1.0E+00 | 1.0E+04 |
| D27 | 2.0E+02 | 1.0E+00 | 2.0E+02 | 1.0E+00 | 1.0E+00 |  |
| D28 | 1.0E+02 | 1.0E+00 | 9.9E+01 | 1.0E+00 | 1.0E+00 |  |

| **Group 4 Mouse 3** | Total  *E. coli* | E. coli (pCT::aph) | Kan^S^  *E. coli* | *E. coli*  (pC-K-307) | Rif^R^ *E. coli*  (pC-K-307) | Rif^R^ Kn^S^  *E. coli* |
| --- | --- | --- | --- | --- | --- | --- |
| D1 | 2.0E+03 |  | 2.0E+03 |  |  |  |
| D2 | 5.0E+03 |  | 5.0E+03 |  |  |  |
| D5 | 3.0E+08 | 3.0E+08 | 3.0E+08 |  |  |  |
| D6 | 2.0E+08 | 1.5E+08 | 2.0E+08 |  |  |  |
| D8 | 7.0E+06 | 1.0E+07 | 7.0E+06 |  |  |  |
| D10 | 2.0E+05 | 1.0E+05 | 2.0E+05 |  |  |  |
| D11 | 3.0E+05 | 3.0E+04 | 2.7E+05 | 2.0E+05 |  |  |
| D13 | 2.0E+06 | 1.0E+04 | 2.0E+06 | 2.0E+06 | 1.5E+02 |  |
| D14 | 4.0E+05 | 5.0E+03 | 4.0E+05 | 3.0E+05 | 2.0E+03 |  |
| D16 | 1.0E+05 | 1.0E+02 | 1.0E+05 | 3.3E+04 | 2.0E+03 |  |
| D18 | 6.0E+04 | 1.0E+00 | 6.0E+04 | 1.0E+04 | 1.0E+03 |  |
| D19 | 5.0E+04 | 1.0E+00 | 5.0E+04 | 1.0E+00 | 1.0E+00 |  |
| D20 | 2.5E+04 | 1.0E+00 | 2.5E+04 | 1.0E+00 | 1.0E+00 |  |
| D22 | 3.0E+04 | 1.0E+00 | 3.0E+04 | 1.0E+00 | 1.0E+00 | 1.0E+02 |
| D24 | 2.0E+04 | 1.0E+00 | 2.0E+04 | 1.0E+00 | 1.0E+00 | 2.00E+02 |
| D27 | 1.0E+02 | 1.0E+00 | 9.9E+01 | 1.0E+00 | 1.0E+00 |  |
| D28 | 2.0E+02 | 1.0E+00 | 2.0E+02 | 1.0E+00 | 1.0E+00 |  |

**Group 5**

| **Group 5**  **Mouse 1** | Total  *E. coli* | *E. coli* (pCT::aph) | *E. coli*  (pC-K-307) | Rif^R^ E. coli (pC-K-307) |
| --- | --- | --- | --- | --- |
| D1 | 2.0E+04 |  |  |  |
| D2 | 2.0E+04 |  |  |  |
| D5 | 2.0E+08 | 2.5E+08 |  |  |
| D6 | 5.0E+08 | 2.3E+08 |  |  |
| D8 | 8.0E+06 | 7.0E+06 |  |  |
| D10 | 3.0E+05 | 2.0E+05 |  |  |
| D11 |  | 2.0E+05 | 3.0E+06 |  |
| D13 |  | 1.0E+05 | 3.0E+06 | 1.0E+02 |
| D14 |  | 2.5E+04 | 6.0E+05 | 1.0E+00 |
| D16 |  | 1.0E+04 | 8.5E+04 | 1.0E+00 |
| D18 |  | 1.0E+04 | 2.5E+04 | 1.0E+00 |
| D19 |  | 2.0E+04 | 2.0E+04 | 1.0E+00 |
| D20 |  | 1.0E+04 | 1.0E+00 | 1.0E+00 |
| D22 |  | 1.0E+03 | 1.0E+00 | 1.0E+00 |
| D24 |  | 3.0E+02 | 1.0E+00 | 1.0E+00 |
| D27 |  | 1.0E+02 |  |  |
| D28 |  | 5.0E+02 |  |  |

| **Group 5 Mouse 2** | Total  *E. coli* | *E. coli* (pCT::aph) | *E. coli*  (pC-K-307) | Rif^R^ *E. coli*  (pC-K-307) |
| --- | --- | --- | --- | --- |
| D1 | 1.0E+04 |  |  |  |
| D2 | 1.5E+04 |  |  |  |
| D5 | 8.0E+07 | 8.0E+07 |  |  |
| D6 | 6.1E+08 | 6.0E+08 |  |  |
| D8 | 6.0E+06 | 5.0E+06 |  |  |
| D10 | 2.0E+06 | 1.0E+06 |  |  |
| D11 |  | 1.0E+05 | 2.0E+06 |  |
| D13 |  | 2.0E+04 | 2.0E+06 | 1.0E+00 |
| D14 |  | 1.0E+04 | 6.0E+05 | 1.0E+02 |
| D16 |  | 1.0E+04 | 4.0E+04 | 1.0E+00 |
| D18 |  | 1.1E+04 | 3.0E+04 | 1.0E+00 |
| D19 |  | 5.1E+03 | 1.0E+04 | 1.0E+00 |
| D20 |  | 7.0E+03 | 1.0E+00 | 1.0E+00 |
| D22 |  | 1.0E+03 | 1.0E+00 | 1.0E+00 |
| D24 |  | 1.0E+03 | 1.0E+00 | 1.0E+00 |
| D27 |  | 2.0E+02 |  |  |
| D28 |  | 1.0E+03 |  |  |

| **Group 5 Mouse 3** | Total  *E. coli* | E. coli (pCT::aph) | *E. coli*  (pC-K-307) | Rif^R^ *E. coli*  (pC-K-307) |
| --- | --- | --- | --- | --- |
| D1 | 6.0E+03 |  |  |  |
| D2 | 8.0E+03 |  |  |  |
| D5 | 2.5E+08 | 2.0E+08 |  |  |
| D6 | 2.3E+08 | 2.3E+08 |  |  |
| D8 | 3.1E+07 | 3.0E+07 |  |  |
| D10 | 1.0E+04 | 1.0E+04 |  |  |
| D11 |  | 1.0E+05 | 1.0E+05 |  |
| D13 |  | 2.0E+04 | 2.0E+06 | 1.0E+00 |
| D14 |  | 3.0E+04 | 2.0E+05 | 1.0E+00 |
| D16 |  | 1.0E+04 | 2.0E+04 | 1.0E+00 |
| D18 |  | 1.0E+04 | 1.0E+04 | 1.0E+00 |
| D19 |  | 1.5E+04 | 2.0E+04 | 1.0E+00 |
| D20 |  | 1.0E+04 | 1.0E+00 | 1.0E+00 |
| D22 |  | 1.0E+03 | 1.0E+00 | 1.0E+00 |
| D24 |  | 7.0E+02 | 1.0E+00 | 1.0E+00 |
| D27 |  | 1.0E+03 |  |  |
| D28 |  | 4.0E+02 |  |  |

**Group 6**

| **Group 6 Mouse 1** | Total  *E. coli* | *E. coli* (pCT::aph) | *E. coli*  (pC-K-307) | Rif^R^ E. coli (pC-K-307) |
| --- | --- | --- | --- | --- |
| D1 | 3.0E+04 |  |  |  |
| D2 | 4.0E+04 |  |  |  |
| D5 | 1.5E+08 | 1.0E+08 |  |  |
| D6 | 1.5E+08 | 1.5E+08 |  |  |
| D8 | 3.0E+06 | 2.0E+06 |  |  |
| D10 | 2.0E+06 | 1.0E+06 |  |  |
| D11 |  | 2.0E+05 | 2.0E+06 | 1.0E+00 |
| D13 |  | 1.0E+04 | 3.0E+05 | 1.0E+02 |
| D14 |  | 2.0E+04 | 3.0E+05 | 1.0E+02 |
| D16 |  | 1.0E+04 | 9.0E+05 | 1.0E+00 |
| D18 |  | 1.0E+03 | 2.0E+06 | 1.0E+00 |
| D19 |  | 1.0E+04 | 3.0E+04 | 1.0E+00 |
| D20 |  | 8.0E+03 | 4.0E+03 | 1.0E+00 |
| D22 |  | 2.0E+03 | 2.0E+02 | 1.0E+00 |
| D24 |  | 1.0E+02 | 1.0E+00 | 1.0E+00 |
| D27 |  | 5.0E+02 |  |  |
| D28 |  | 1.0E+03 |  |  |

| **Group 6 Mouse 2** | Total  *E. coli* | *E. coli* (pCT::aph) | *E. coli*  (pC-K-307) | Rif^R^ *E. coli*  (pC-K-307) |
| --- | --- | --- | --- | --- |
| D1 | 1.0E+03 |  |  |  |
| D2 | 5.0E+03 |  |  |  |
| D5 | 1.5E+08 | 1.5E+08 |  |  |
| D6 | 6.0E+08 | 5.0E+07 |  |  |
| D8 | 3.0E+06 | 3.0E+05 |  |  |
| D10 | 1.5E+05 | 1.0E+05 |  |  |
| D11 |  | 1.0E+05 | 2.0E+05 | 1.0E+00 |
| D13 |  | 1.0E+03 | 1.0E+06 | 1.0E+00 |
| D14 |  | 1.2E+04 | 6.0E+05 | 1.0E+00 |
| D16 |  | 3.0E+03 | 7.0E+06 | 1.0E+00 |
| D18 |  | 3.0E+03 | 3.0E+06 | 1.0E+00 |
| D19 |  | 2.0E+04 | 1.0E+05 | 1.0E+00 |
| D20 |  | 1.0E+04 | 2.0E+04 | 1.0E+00 |
| D22 |  | 1.0E+04 | 3.0E+02 | 1.0E+00 |
| D24 |  | 2.2E+03 | 1.0E+00 | 1.0E+00 |
| D27 |  | 3.0E+02 |  |  |
| D28 |  | 2.0E+02 |  |  |

| **Group 6 Mouse 3** | Total  *E. coli* | E. coli (pCT::aph) | *E. coli*  (pC-K-307) | Rif^R^ *E. coli*  (pC-K-307) |
| --- | --- | --- | --- | --- |
| D1 | 4.0E+04 |  |  |  |
| D2 | 1.0E+04 |  |  |  |
| D5 | 2.7E+08 | 2.0E+08 |  |  |
| D6 | 1.0E+08 | 1.0E+08 |  |  |
| D8 | 3.5E+06 | 2.0E+06 |  |  |
| D10 | 2.0E+04 | 1.0E+04 |  |  |
| D11 |  | 3.0E+05 | 1.0E+05 | 1.0E+00 |
| D13 |  | 3.0E+03 | 6.0E+05 | 1.0E+00 |
| D14 |  | 1.0E+04 | 5.6E+05 | 1.0E+02 |
| D16 |  | 2.0E+04 | 1.2E+07 | 2.0E+02 |
| D18 |  | 1.0E+04 | 2.0E+06 | 1.0E+00 |
| D19 |  | 1.0E+04 | 2.0E+06 | 1.0E+00 |
| D20 |  | 1.0E+05 | 1.0E+04 | 1.0E+00 |
| D22 |  | 1.0E+05 | 1.0E+03 | 1.0E+00 |
| D24 |  | 7.0E+03 | 1.0E+00 | 1.0E+00 |
| D27 |  | 2.0E+03 |  |  |
| D28 |  | 1.0E+03 |  |  |
