# Supplementary figures and images for: Potentiation of curing by a broad-host-range self-transmissible vector for displacing resistance plasmids to tackle AMR
Source: PLoS One. 2020 Jan 15;15(1):e0225202. doi: 10.1371/journal.pone.0225202 (PMC6961859; doi:10.1371/journal.pone.0225202)

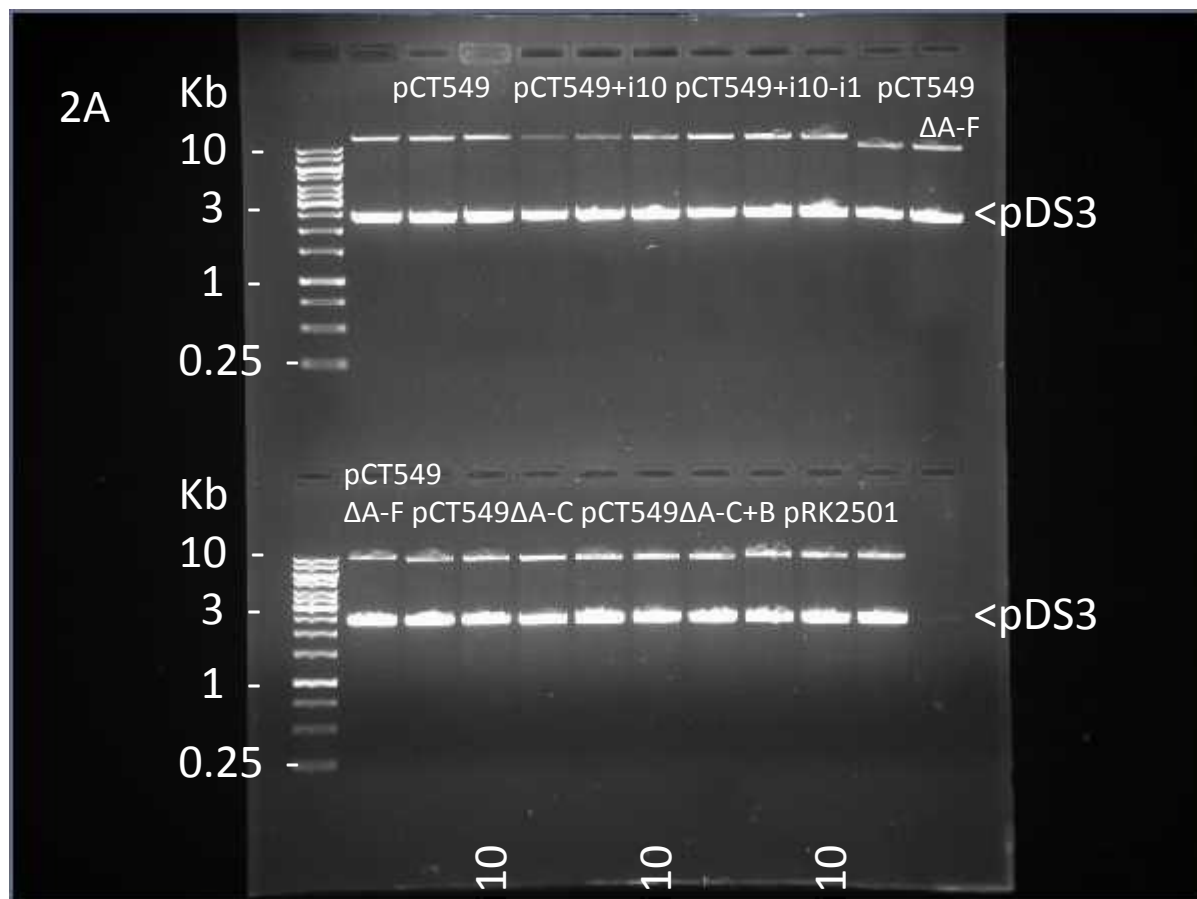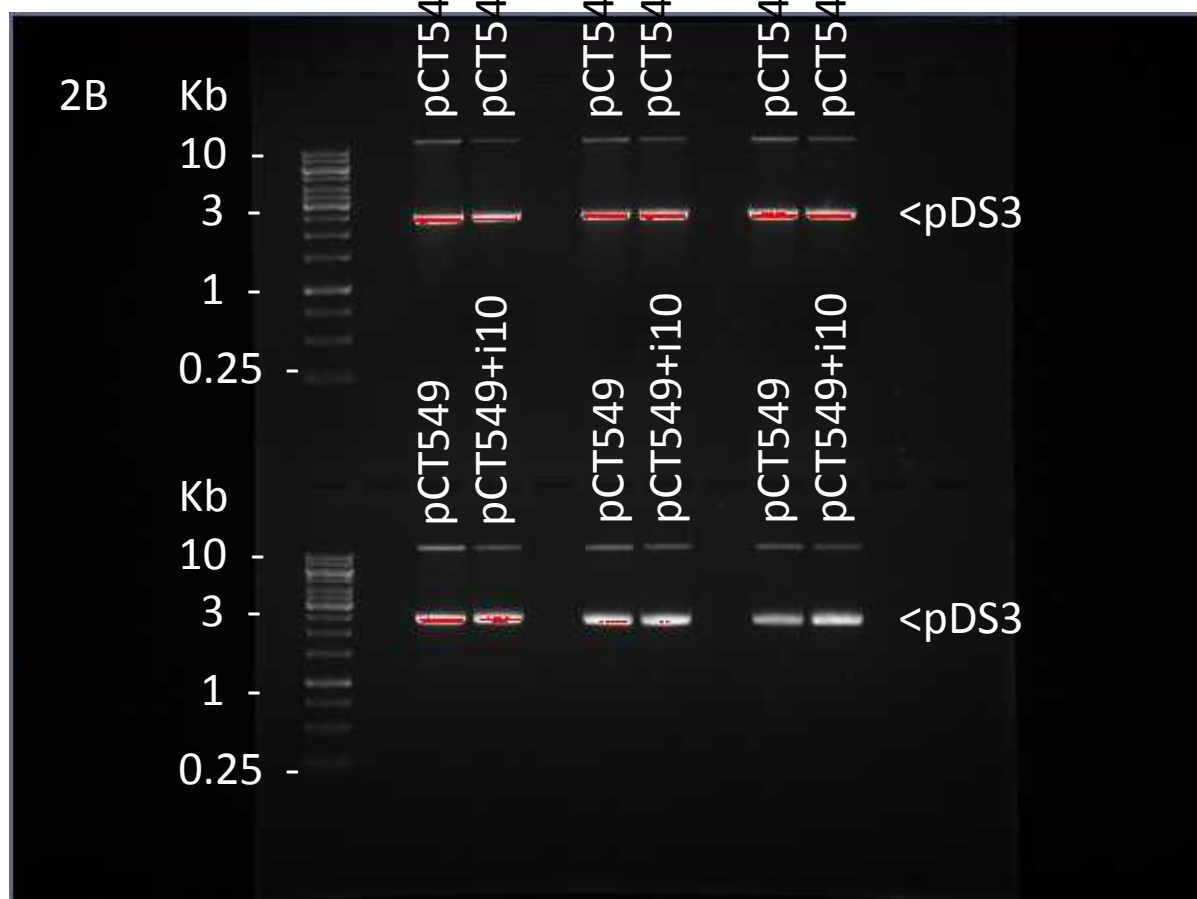

Supplement: S1 Raw Images — (PDF) [file pone.0225202.s014.pdf]
